# Synthesis and Evaluation of ^11^C-Labeled Triazolones as Probes for Imaging Fatty Acid Synthase Expression by Positron Emission Tomography

**DOI:** 10.3390/molecules27051552

**Published:** 2022-02-25

**Authors:** James M. Kelly, Thomas M. Jeitner, Nicole N. Waterhouse, Wenchao Qu, Ethan J. Linstad, Banafshe Samani, Clarence Williams, Anastasia Nikolopoulou, Alejandro Amor-Coarasa, Stephen G. DiMagno, John W. Babich

**Affiliations:** 1 Molecular Imaging Innovations Institute (MI3), Department of Radiology, Weill Cornell Medicine, New York, NY 10065, USA; tmj4001@med.cornell.edu (T.M.J.); clw2012@med.cornell.edu (C.W.J.); ann2010@med.cornell.edu (A.N.); alejandro.amor@einsteinmed.edu (A.A.-C.); jwbabich@gmail.com (J.W.B.); 2 Citigroup Biomedical Imaging Center, Weill Cornell Medicine, New York, NY 10021, USA; niw2014@med.cornell.edu (N.N.W.); weq2002@med.cornell.edu (W.Q.); 3 Departments of Medicinal Chemistry & Pharmacognosy and Chemistry, University of Illinois-Chicago, Chicago, IL 60612, USA; ethanl@uic.edu (E.J.L.); bsaman4@uic.edu (B.S.); sdimagno@uic.edu (S.G.D.); 4 Department of Chemistry, University of Nebraska-Lincoln, Lincoln, NE 68588, USA; 5 Sandra and Edward Meyer Cancer Center, Weill Cornell Medicine, New York, NY 10065, USA

**Keywords:** fatty acid synthase, cancer metabolism, positron emission tomography, carbon-11

## Abstract

Cancer cells require lipids to fulfill energetic, proliferative, and signaling requirements. Even though these cells can take up exogenous fatty acids, the majority exhibit a dependency on de novo fatty acid synthesis. Fatty acid synthase (FASN) is the rate-limiting enzyme in this process. Expression and activity of FASN is elevated in multiple cancers, where it correlates with disease progression and poor prognosis. These observations have sparked interest in developing methods of detecting FASN expression in vivo. One promising approach is the imaging of radiolabeled molecular probes targeting FASN by positron emission tomography (PET). However, although [^11^C]acetate uptake by prostate cancer cells correlates with FASN expression, no FASN-specific PET probes currently exist. Our aim was to synthesize and evaluate a series of small molecule triazolones based on GSK2194069, an FASN inhibitor with IC_50_ = 7.7 ± 4.1 nM, for PET imaging of FASN expression. These triazolones were labeled with carbon-11 in good yield and excellent radiochemical purity, and binding to FASN-positive LNCaP cells was significantly higher than FASN-negative PC3 cells. Despite these promising characteristics, however, these molecules exhibited poor in vivo pharmacokinetics and were predominantly retained in lymph nodes and the hepatobiliary system. Future studies will seek to identify structural modifications that improve tumor targeting while maintaining the excretion profile of these first-generation ^11^C-methyltriazolones.

## 1. Introduction

Increased lipogenesis is a phenotypic hallmark of many cancer cells [1,2]. Fatty acids support a number of essential processes in cancer, including proliferation, energy, oncogenic signaling pathways, and resistance to therapy [1,3]. The fatty acid pool in cancer cells is fed by both de novo fatty acid synthesis and the uptake of exogenous fatty acids [4]. However, the majority of cancer cells overexpress lipogenic enzymes, including fatty acid synthase (E.C. 2.3.1.85; FASN) and exhibit a dependency on de novo fatty acid synthesis [5]. FASN synthesizes palmitate from acetyl-CoA and malonyl-CoA, using NADPH as a reducing equivalent [2], and is the rate-limiting enzyme of de novo fatty acid synthesis [6]. The products of de novo fatty acid synthesis in cancer cells are predominantly esterified to phospholipid aggregates that participate in signal transduction, intracellular trafficking, and cell migration [7]. FASN is also proposed to support post-translational modification and provide palmitate as a source of fuel [8]. FASN expression is regulated by the PI3K/Akt axis [9], which is frequently dysregulated in cancer [10]. Consequently, expression and activity of FASN is elevated in multiple cancers [11,12,13,14,15], where it correlates with disease progression and poor prognosis [13,16,17].

In contrast to cancerous cells, FASN expression in normal tissue is typically low [5]. This facilitates a therapeutic strategy based on inhibition of FASN activity. FASN exists as a homodimer comprised of identical subunits of seven domains, many of which have been targeted by pharmacological inhibitors [2,18]. Early studies using broader spectrum inhibitors targeting specific domains of FASN demonstrated decreased viability of cancer cells and restrained tumor growth in xenograft models as a consequence of FASN inhibition [19,20,21,22,23,24,25,26] but these compounds were limited by poor solubility, poor bioavailability, and off-target toxicity [2,18,27]. More recently, multiple classes of high affinity small molecule inhibitors have been developed and advanced into preclinical [28,29,30,31,32] and clinical evaluation [33,34].

In spite of the prevalence of small molecule FASN inhibitor platforms, no FASN-specific agent exists for in vivo imaging of FASN expression by positron emission tomography (PET) [34]. Our aims were to develop a family of ^11^C-labeled FASN inhibitors based on high affinity triazolone-containing compounds related to GSK2194069 [35,36] and to evaluate these compounds in prostate cell lines and xenograft models characterized by high and low FASN expression. We report the synthesis of three [^11^C]methyltriazolones that are rapidly taken up by lymph node carcinoma of the prostate (LNCaP) cells in an FASN-specific manner, but are limited in their imaging utility by poor in vivo pharmacokinetics. These compounds may pave the way for radiolabeled FASN inhibitors with better pharmacokinetics in the future.

## 2. Results

### 2.1. Lead Compound Identification

**Figure 1:**
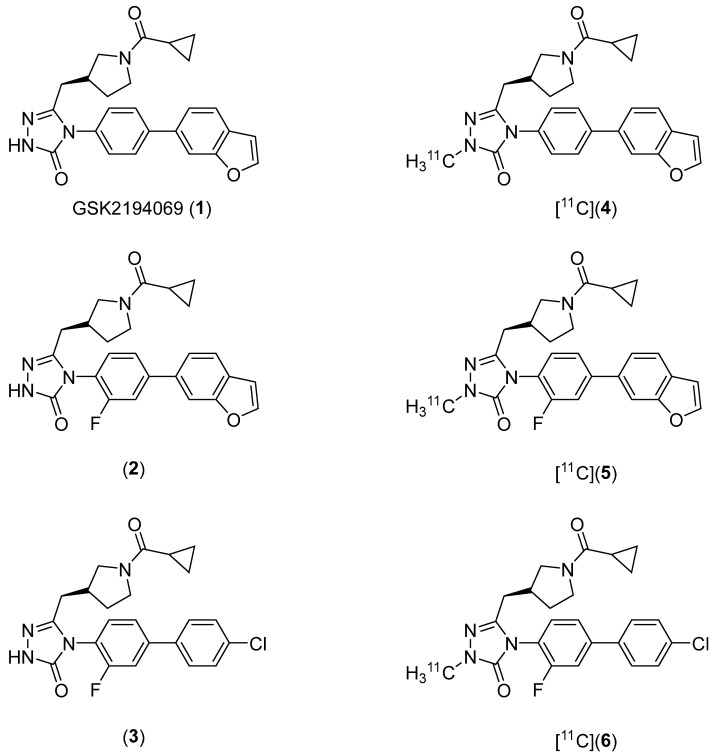
Target compounds. Fluorine-containing compounds **2** and **3**, and their derivatives [^11^C]**5** and [^11^C]**6**, were selected to allow the possibility of ^18^F-fluorination as an alternative labeling strategy.

Our strategy for developing ^11^C-labeled FASN inhibitors was to *N*-alkylate triazolone FASN inhibitors with [^11^C]CH_3_I or [^11^C]CH_3_OTf. This approach was based on the observation that *N*-methylation of the triazolone moiety of GSK2194069 (**1**), a commercially-available, high affinity FASN ligand, does not substantially decrease FASN affinity (10 nM vs. 20 nM) [35]. We selected two fluorine-containing triazolones (**2** and **3**) from a library of previously published library of GSK2194069 analogues [35] for this purpose since they could be labeled with fluorine-18 as an alternative to carbon-11 should our initial studies prove successful. Fluorine-18 has superior physical properties (e.g., higher positron branching, lower positron E_max_) for PET imaging than carbon-11, but the chemistry of fluoride incorporation on electron-rich aromatic rings is challenging. Our target compounds are shown in Figure 1.

### 2.2. Radiosynthesis

**Figure 2:**
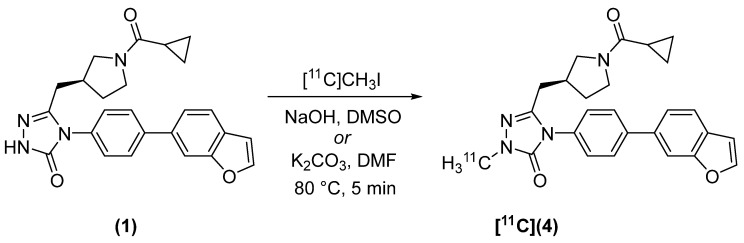
Radiosynthesis of [^11^C](**4**).

The compounds were synthesized by base-catalyzed alkylation of the triazolone precursor. Each compound was isolated in greater than 97% radiochemical purity (Figures S1–S3).

(*S*)-[*methyl*-^11^C]4-(4-(Benzofuran-6-yl)phenyl)-5-((1-(cyclopropanecarbonyl)pyrrolidin-3-yl)methyl)-2-methyl-2,4-dihydro-3*H*-1,2,4-triazol-3-one ([^11^C]**4**) was synthesized from GSK2194069 (Figure 2) in 44 ± 2 min from end of bombardment (EOB). Production and delivery of [^11^C]CH_3_I was accomplished in 14 ± 1 min, and the synthesis of [^11^C]**4** was therefore completed in approximately 30 min from delivery of [^11^C]CH_3_I. The radiochemical yield of [^11^C]**4**, decay-corrected (dc) to the delivery of [^11^C]CH_3_I, was 46.8 ± 9.0% (*n* = 12) when NaOH was used as base and DMSO as the solvent. The molar activity, A_m_, was 420 ± 320 GBq/µmol. When K_2_CO_3_/DMF were used, [^11^C]**4** was isolated in 52.5 ± 1.5% decay-corrected radiochemical yield (dc RCY) (*n* = 2) and A_m_ = 240 GBq/µmol.

**Figure 3:**
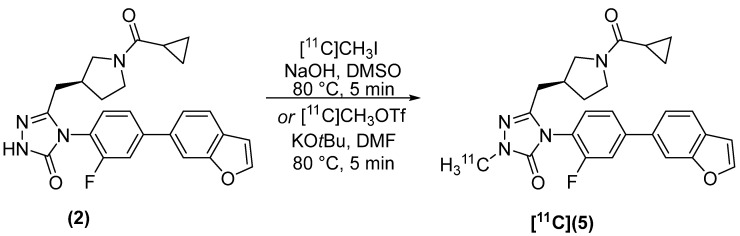
Radiosynthesis of [^11^C](**5**).

**Figure 4:**
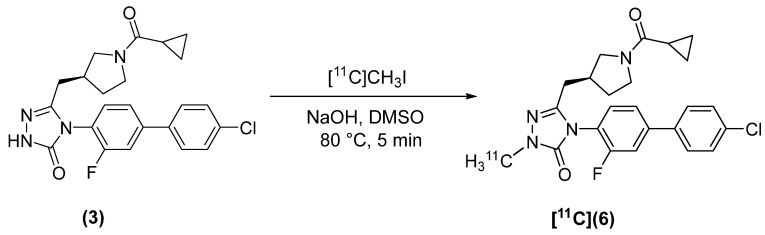
Radiosynthesis of [^11^C](**6**).

The synthesis of (*S*)-[*methyl*-^11^C]4-(4-(benzofuran-6-yl)-2-fluorophenyl)-5-((1-(cyclopropanecarbonyl)pyrrolidin-3-yl)methyl)-2-methyl-2,4-dihydro-3*H*-1,2,4-triazol-3-one ([^11^C]**5**) was accomplished by two strategies. Our first method employed [^11^C]CH_3_I as the alkylating agent (Figure 3) and resulted in a dc RCY, corrected to delivery of [^11^C]CH_3_I, of 43.2 ± 17.8% (*n* = 3) and A_m_ = 322 ± 96 GBq/µmol. Synthesis time was 48 ± 2 min from EOB. Alkylation of (**2**) was also accomplished using [^11^C]CH_3_OTf in 50 ± 1 min from EOB, of which 16 ± 1 min were required to produce and deliver [^11^C]CH_3_OTf. Under these reaction conditions, the dc RCY, corrected to delivery of [^11^C]CH_3_OTf, was 65.3 ± 18.5% (*n* = 3) and A_m_ = 1280 GBq/µmol.

(*S*)-[*methyl*-^11^C]-4-(4′-Chloro-3-fluoro-[1,1′-biphenyl]-4-yl)-5-((1-(cyclopropanecarbonyl)pyrrolidin-3-yl)methyl)-2-methyl-2,4-dihydro-3H-1,2,4-triazol-3-one ([^11^C]**6**) was synthesized using [^11^C]CH_3_I (Figure 4). The final product was isolated in 61.8 ± 15.5% dc RCY (*n* = 3) and A_m_ = 49 ± 27 GBq/µmol (*n* = 3). Synthesis time was 33 ± 6 min from EOB.

### 2.3. In Vitro Cell Binding

**Figure 5:**
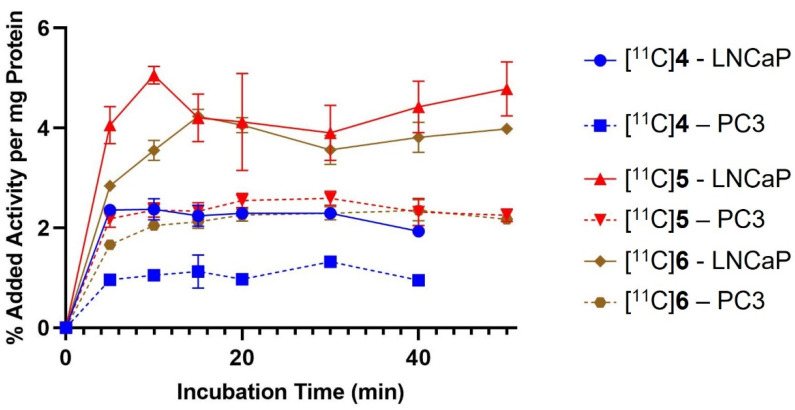
Time course binding of [^11^C]**4** (blue), [^11^C]**5** (red), and [^11^C]**6** (brown) to LNCaP cells or PC3 cells incubated at 37 °C for up to 50 min. The counts were corrected for decay and normalized to protein content.

The activity of the probes was determined against LNCaP cells, which highly express FASN (Figure S4), and PC3 prostate cancer cells, which express FASN at much lower levels and served as our negative controls. Each of the compounds exhibited higher binding to LNCaP cells than to PC3 cells after the harvested counts were normalized to protein content. Binding was characterized by a rapid initial rate of uptake, followed by a period of equilibrium (Figure 5). Peak cell binding was reached after 10–15 min and was two-fold higher in LNCaP cells. Efflux of radioactivity was negligible up to 50 min after addition of activity. Peak binding of [^11^C]**5** and [^11^C]**6** to LNCaP cells was comparable (4.23 ± 0.14 vs. 5.06 ± 0.18% added activity), while peak binding of [^11^C]**4** was slightly lower (2.37 ± 0.21% added activity). Binding of [^11^C]**5** to LNCaP cells was significantly higher than PC3 cells (*p* = 0.03).

### 2.4. In Vitro Growth Inhibition Assays

**Figure 6:**
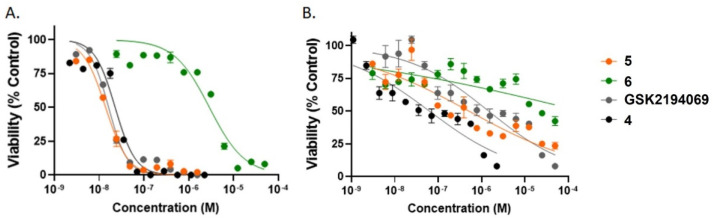
Growth inhibition of (**A**) LNCaP cells or (**B**) PC3 cells following treatment with GSK2194069 (gray), or the non-radioactive standards **4** (black), **5** (orange), and **6** (green) for 4 d. Studies were performed in triplicate.

The ability of the compounds to inhibit the growth of LNCaP and PC3 cells was determined for ligand concentrations ranging from 1 nM to 100 µM (Figure 6). The EC_50_ in LNCaP cells of GSK2194069 is 16 nM. By comparison, the EC_50_ of GSK2194069 in A549 cells, another cell line with high FASN expression, is 15 ± 0.5 nM [36]. Analogues **5** and **4** substantially reduced the viability of LNCaP cells, with EC_50_ = 14 nM and EC_50_ = 22 nM, respectively. By contrast, compound **6** did not inhibit LNCaP cells as successfully, with EC_50_ = 2.9 μM.

Each of the compounds preferentially kills LNCaP cells as compared to PC3 cells. The effectiveness in PC3 cells is reduced by a factor of 90 for GSK2194069 (EC_50_ = 1.5 µM) and a factor of nearly 40 for **5** (EC_50_ = 551 nM) (Figure S4).

### 2.5. MicroPET/CT Imaging

**Figure 7:**
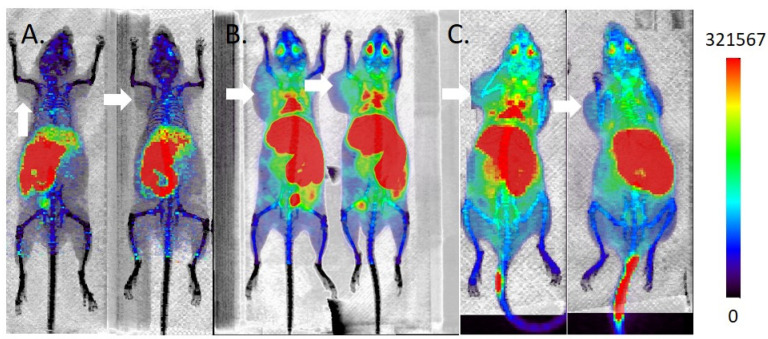
Maximum intensity projection microPET/CT images of ^11^C-labeled triazolones in male inbred athymic nu/nu mice bearing LNCaP xenografts (*n* > 4 per compound). Mice were imaged in groups of 4, with 2 representative examples shown per compound. Tumors are indicated with white arrows. The mice were administered (**A**) 8–10 MBq [^11^C]**4** in 10% EtOH/saline; (**B**) 9–11 MBq [^11^C]**5** in 10% EtOH/saline; (**C**) 9–11 MBq [^11^C]**6** in 10% EtOH/saline.

In spite of promising in vitro activities in LNCaP cells, none of the ^11^C-labeled FASN inhibitors accumulates in LNCaP xenograft tumors. [^11^C]**4** distributes to the hepatobiliary system (Figure 7A). In addition, accumulation in thymus is evident at early time points but clears by 60 min p.i. [^11^C]**5** predominantly accumulates in the liver and intestines (Figure 7B), with uptake in lungs at early time points evident. In addition, uptake is evident in the brachial, cervical, and lumbar lymph nodes. The tumor is indistinguishable from background. To account for the possibility that the uptake in liver and nodes is due to self-aggregation of the lipophilic probe, imaging was performed using a pre-mixed solution of [^11^C]**5** and Captisol^®^ (Ligand, San Diego, CA, USA), a modified cyclodextrin solubilizing agent [37]. There was no apparent change in the tissue distribution (data not shown). [^11^C]**6** distributes similarly to both [^11^C]**4** and [^11^C]**5** (Figure 7C). FASN expression was confirmed in the xenografted tumors by Western blot (Figure S4).

## 3. Discussion

In contrast to many cancers, prostate cancer exhibits highly variable uptake of [^18^F]FDG [38]. This variability likely reflects the biological and clinical heterogeneity of disease [39]. [^18^F]FDG uptake has prognostic value, particularly in metastatic prostate cancer [40,41,42], but uptake in androgen-sensitive disease is generally low [43]. [^11^C]Choline and [^18^F]fluorocholine exploit upregulation of choline metabolism in prostate cancer, which leads to increased phosphatidylcholine levels and turnover [44,45]. Similarly to [^18^F]FDG PET, [^11^C]choline and [^18^F]fluorocholine PET suffer from diminished sensitivity in primary cancer, but may be of greater value in detecting recurrent disease [46]. Sensitive detection of prostate cancer by metabolic imaging therefore requires other metabolic pathways to be considered.

Lipid metabolism is upregulated even at early stages of prostate cancer [47], rendering this pathway an attractive target for diagnostic and prognostic imaging. FASN catalyzes the de novo synthesis of long-chain fatty acids and is an important regulator of lipid metabolism [48]. In spite of the emerging importance of FASN as a marker of tumor aggressiveness [11], a possible pharmacological target [31,32], and a prognostic indicator [11,49], there are no probes for direct in vivo PET imaging of FASN expression. [^11^C]Acetate is proposed to be a probe for FASN on the basis that its uptake correlates with FASN expression in multiple prostate cancer cell lines [50,51]. More recent studies in hepatocellular carcinoma cell lines do not find similar correlation [52]. This discrepancy can be explained by the fact that [^11^C]acetate uptake is only an indirect measure of lipid synthesis. [^11^C]Acetate is thought to be taken up by monocarboxylate transporters [53]. In the cell, [^11^C]acetate may be oxidized in mitochondria, resulting in the liberation of [^11^C]CO_2_ [54], or act as a substrate for Acetyl-CoA synthetase [55]. As [^11^C]CO_2_ rapidly effluxes from cells and tissue, retention of radioactivity in tumors is likely to be due to formation of acetyl-CoA [54]. However, acetyl-CoA is not only a building block for fatty acid synthesis, but for sterols, ketone bodies, and the TCA cycle as well [56]. In this light, increased uptake of [^11^C]acetate in tumors may indicate upregulation of alternative mechanisms to glycolysis for preserving the pool of acetyl-CoA [57].

[^18^F]Fluoroacetate is a potential analogue of [^11^C]acetate on the basis of structural similarity [58], but in vivo defluorination is significant in preclinical models [58,59], and [^18^F]fluoroacetate does not appear to be a functional analogue of [^11^C]acetate [60]. 2-[^18^F]Fluoropropionic acid is another short chain fatty acid analogue that shows good uptake and retention in prostate cancer [60] and liver cancer xenografts [61,62]. However, to date, the significance of 2-[^18^F]fluoropropionic acid tumor uptake remains poorly understood. Uptake of 2-[^18^F]fluoropropionic acid appears to correlate with FASN expression [62], but the precise biochemical pathways and cellular entities targeted by this tracer have yet to be elucidated.

In contrast to prior approaches to imaging FASN, we chose small molecule inhibitors of FASN as parent structures for radiotracer development. We reasoned that these radiotracers would be specific for FASN expression. Our compound library was based on one of the most potent and selective inhibitors of FASN reported to date. GSK2194069 targets the β-ketoacyl reductase domain of human FASN and inhibits the enzyme with IC_50_ < 10 nM [36]. Methylation of the triazolone moiety of GSK2194069 (**4**; Figure 1) retains its potency, with IC_50_ = 20 nM [35]. Similarly, introduction of a fluorine atom at the 2-position of the phenyl ring, results in compounds with a potency as high as 3 nM depending on the selection of (hetero)aromatic substituent at the 4-position of the phenyl ring. Our structural modifications, which consisted of the addition of a fluorine atom to **4** (**5**; Figure 1), and substitution of a 4-chlorophenyl moiety for the benzofuran moiety of **5** (**6**; Figure 1), had variable effects on potency. Analogue **5** retained the potency of GSK2194069 in LNCaP cells, but the potency of **6** was substantially compromised.

Our quantification of FASN expression by Western blot confirms high expression in LNCaP cells and much lower expression in PC3 cells (Figure S4), in agreement with literature reports [50]. In this light, the greater binding seen in LNCaP cells is consistent with FASN targeting. The difference in binding between the two cell lines is approximately two-fold but binding to both cell lines is relatively low when expressed as a percentage of the added dose. It is possible that the difference would be accentuated at higher levels of cell binding. When LNCaP cells were cultured for longer than 48 h, we achieved greater total cell binding (17% vs. 7% added activity of [^11^C]**5** at peak binding; Figure S6), but cells no longer grew as a monolayer. Consequently, we adjusted our assay conditions to increase reproducibility at the expense of total cell binding.

Notwithstanding our promising findings in vitro, the tumor targeting of [^11^C]**4**, [^11^C]**5**, and [^11^C]**6** in vivo was poor. Given that we did not observe a loss in FASN expression in the tumors relative to the parent cell line (Figure S4), we investigated alternative explanations. Alkylation of triazolones on the oxygen atom under basic conditions has been reported [63]. In the absence of reference standards for *O*-methylation, we could not be certain that our analytical method could distinguish between the *N*-[^11^C]methyl and *O*-[^11^C]methyl compounds. Therefore, we explored alternative radiolabeling strategies, including variation of base (NaOH, K_2_CO_3_, KO*t*Bu), solvent (DMF, DMSO), reaction temperature (25 °C, 80 °C), and alkylating agent ([^11^C]CH_3_I, [^11^C]CH_3_OTf). Small differences in decay corrected radiochemical yield were evident, but the purified final products behaved identically in analytical assays and in vivo imaging studies. These findings led us to conclude that the poor tumor targeting is a consequence of poor pharmacokinetics. Our images indicated high retention of signal in the hepatobiliary pathway. We did not observe significant renal clearance, and consequently activity in the bladder was low. This contrasts with [^18^F]FDG, for which accumulation of activity in the bladder can obscure the primary tumor in the prostate gland. Our next generation FASN ligands will seek to retain the excretion profile of these first-generation compounds while improving the tumor targeting.

## 4. Materials and Methods

### 4.1. Synthesis of Precursors

(*S*)-4-(4-(Benzofuran-6-yl)phenyl)-5-((1-(cyclopropanecarbonyl) pyrrolidin-3-yl)methyl)-2,4-dihydro-3*H*-1,2,4-triazol-3-one (GSK2194069) was purchased from Tocris Bioscience (Minneapolis, MN, USA) and used without further purification. The remaining precursors and non-radioactive standards were synthesized according to published procedures [35]. The characterization of final compounds is described herein, with full experimental details available in the Supporting Information Scheme S1.

(*S*)-4-(4-(Benzofuran-6-yl)phenyl)-5-((1-(cyclopropanecarbonyl)pyrrolidin-3-yl)methyl)-2-methyl-2,4-dihydro-3*H*-1,2,4-triazol-3-one (**4**).

^1^H-NMR (CDCl_3_, 500 MHz, 25 °C): δ 7.83 (d, *J* = 1.5 Hz, 1H), 7.76 (d, *J* = 8.5 Hz, 2H), 7.71 (d, *J* = 2.5 Hz, 1H), 7.61 (d, *J* = 8.5 Hz, 1H), 7.55 (dd, *J* = 8.5 Hz, 2.0 Hz, 1H), 7.38 (d, *J* = 8.0 Hz, 2H), 6.86 (d, *J* = 2.0 Hz, 0.5 Hz, 1H), 3.85 (m, 1H), 3.70 (m, 1H), 3.53 (m, 1H), 3.18 (m, 1H), 3.55 (s, 3H), 2.64–2.42 (br m, 2.5H), 2.26–2.15 (br m, 1H), 2.10–2.02 (m, 0.5H), 1.77–1.68 (m, 0.5H), 1.61–1.56 (m, 1.5H), 1.02–0.96 (m, 2H), 0.79–0.74 (m, 2H). ^13^C-NMR (CDCl_3_, 150 MHz, 25 °C): δ 172.3, 154.8, 153.5, 145.9, 142.8, 137.2, 135.1, 131.5, 128.9, 128.8, 128.1, 127.4, 127.3, 123.9, 119.9, 111.8, 106.8, 51.8, 45.3, 42.8, 32.5, 29.7, 22.8, 12.2, 7.6, 7.5. LCMS calc. for C_26_H_26_N_4_O_3_ [M + H]^+^: 443.20. Found: 443.24. [α]^20^_D_ = −8.04 ± 0.59° (CH_2_Cl_2_).

(*R*)-4-(4-(Benzofuran-6-yl)-2-fluorophenyl)-5-((1-(cyclopropanecarbonyl)pyrrolidin-3-yl)methyl)-2,4-dihydro-3*H*-1,2,4-triazol-3-one (**2**).

^1^H-NMR (CDCl_3_, 400 MHz, 25 °C): δ 10.24 (br s, 0.5H), 10.12 (br s, 0.5H), 7.80 (br s, 1H), 7.69 (br s, 1H), 7.60 (dd, *J* = 8.5 Hz, 2.8 Hz, 1H), 7.56–7.50 (m, 3H), 7.42 (dt, *J* = 11.7 Hz, 2.0 Hz, 1H), 6.84 (br s, 1H), 3.96–3.92 (m, 0.5H), 3.78–3.69 (m, 1H), 3.66–3.58 (m, 1H), 3.42–3.35 (m, 0.5H), 3.30–3.26 (m, 0.5H), 3.10–3.06 (m, 0.5H), 2.74–2.64 (m, 0.5H), 2.64–2.42 (br m, 2.5H), 2.26–2.15 (br m, 0.5H), 2.10–2.02 (m, 0.5H), 1.77–1.68 (m, 0.5H), 1.62–1.54 (m, 1.5H), 1.02–0.93 (m, 2H), 0.78–0.69 (m, 2H). ^13^C-NMR (CDCl_3_, 100 MHz, 25 °C): δ 172.4, 159.1, 156.6, 155.3, 155.2, 155.1, 146.6, 146.3, 146.2, 146.0, 145.93, 145.90, 145.8, 134.02, 133.95, 130.1, 130.0, 128.39, 128.37, 124.4, 123.9, 120.1, 118.6, 118.52, 118.48, 118.39, 116.1, 115.9, 112.13, 112.09, 106.9, 51.8, 51.2, 51.0, 45.9, 45.3, 36.0, 34.3, 31.6, 30.5, 29.5, 29.3, 12.6, 12.4, 7.8, 7.7, 7.6. ^19^F-NMR (CDCl_3_, 376 MHz, 25 °C): δ-120.2 (br s, 1F). HRMS (ESI+) calculated for C_25_H_23_FN_4_O_3_: 446.1754. Found: 446.1685. [α]^20^_D_ = −8.35 ± 0.30° (CH_2_Cl_2_).

(*R*)-4-(4-(Benzofuran-6-yl)-2-fluorophenyl)-5-((1-(cyclopropanecarbonyl)pyrrolidin-3-yl)methyl)-2-methyl-2,4-dihydro-3*H*-1,2,4-triazol-3-one (**5**).

^1^H-NMR (CDCl_3_, 400 MHz, 25 °C): δ 7.80 (s, 1H), 7.69 (s, 1H), 7.59 (d, *J* = 8.4 Hz, 1H), 7.54–7.48 (m, 3H), 7.39 (dd, *J* = 8.7 Hz, 7.7 Hz, 1H), 6.84 (s, 1H), 3.94–3.90 (m, 0.5H), 3.79–3.69 (m, 1H), 3.65–3.56 (m, 1H), 3.52 (d, *J* = 6.9 Hz, 3H), 3.42–3.35 (m, 0.5H), 3.28–3.24 (m, 0.5H), 3.08–3.04 (m, 0.5H), 2.72–2.63 (m, 0.5H), 2.63–2.45 (br m, 2.5H), 2.26–2.15 (br m, 0.5H), 2.10–2.02 (m, 0.5H), 1.75–1.66 (m, 0.5H), 1.60–1.51 (m, 1.5H), 1.05–0.92 (m, 2H), 0.78–0.69 (m, 2H). ^13^C-NMR (CDCl_3_, 100 MHz, 25 °C): δ 172.3, 159.1, 156.6, 155.2, 146.24, 146.20, 145.84, 145.76, 145.73, 145.66, 144.5, 134.03, 133.98, 130.1, 130.0, 128.4, 124.3, 123.9, 120.1, 119.1, 119.02, 118.95, 118.89, 116.0, 115.9, 112.09, 112.07, 106.9, 51.8, 51.2, 51.0, 45.9, 45.3, 36.0, 34.3, 32.7, 31.6, 30.5, 29.5, 29.3, 12.6, 12.4, 7.8, 7.7, 7.6. ^19^F-NMR (CDCl_3_, 376 MHz, 25 °C): δ-120.3 (br s, 1F). HRMS (ESI+) calculated for C_26_H_25_FN_4_O_3_: 460.1911. Found: 460.1846. [α]^20^_D_ = −2.99 ± 0.28° (CH_2_Cl_2_).

(*R*)-4-(4′-Chloro-3-fluoro-[1,1′-biphenyl]-4-yl)-5-((1-(cyclopropanecarbonyl)pyrrolidin-3-yl)methyl)-2,4-dihydro-3H-1,2,4-triazol-3-one (**3**).

^1^H-NMR (CDCl_3_, 400 MHz, 25 °C): δ 10.94 (br s, 1H), 7.50 (dd, *J* = 8.5 Hz, 1.6 Hz, 2H), 7.47–7.39 (m, 5H), 3.93–3.89 (m, 0.5H), 3.75–3.67 (m, 1H), 3.63–3.53 (m, 1H), 3.40–3.33 (m, 0.5H), 3.27–3.23 (m, 0.5H), 3.07–3.02 (m, 0.5H), 2.70–2.63 (m, 0.5H), 2.63–2.40 (m, 2.5H), 2.23–2.14 (m, 0.5H), 2.08–2.00 (m, 0.5H), 1.73–1.64 (m, 0.5H), 1.59–1.49 (m, 1.5H), 1.01–0.91 (m, 2H), 0.78–0.69 (m, 2H). ^13^C-NMR (CDCl_3_, 100 MHz, 25 °C): δ 172.5, 159.1, 156.5, 155.15, 155.09, 146.2, 144.1, 144.0, 143.9, 137.2, 137.1, 135.0, 134.9, 130.3, 130.2, 129.41, 129.38, 128.5, 123.9, 119.31, 119.23, 119.18, 119.10, 115.7, 115.5, 51.8, 51.2, 45.9, 45.3, 35.9, 34.3, 31.5, 30.4, 29.4, 29.2, 12.6, 12.3, 7.74, 7.67, 7.57. ^19^F-NMR (CDCl_3_, 376 MHz, 25 °C): δ-119.7 (br s, 1F). HRMS (ESI+) calculated for C_23_H_22_ClFN_4_O_2_: 440.1415. Found: 440.1339. [α]^20^_D_ = −9.37 ± 0.34° (CH_2_Cl_2_).

(*R*)-4-(4′-Chloro-3-fluoro-[1,1′-biphenyl]-4-yl)-5-((1-(cyclopropanecarbonyl)pyrrolidin-3-yl)methyl)-2-methyl-2,4-dihydro-3*H*-1,2,4-triazol-3-one (**6**).

^1^H-NMR (CDCl_3_, 400 MHz, 25 °C): δ 7.52 (br d, *J* = 8.4 Hz, 2H), 7.47–7.39 (m, 5H), 3.93–3.89 (m, 0.5H), 3.75–3.67 (m, 1H), 3.63–3.56 (m, 1H), 3.52 (s, 1.5H), 3.51 (s, 1.5H), 3.40–3.33 (m, 0.5H), 3.27–3.23 (m, 0.5H), 3.07–3.02 (m, 0.5H), 2.70–2.63 (m, 0.5H), 2.63–2.40 (m, 2.5H), 2.23–2.14 (m, 0.5H), 2.08–2.00 (m, 0.5H), 1.73–1.64 (m, 0.5H), 1.59–1.49 (m, 1.5H), 1.01–0.91 (m, 2H), 0.78–0.69 (m, 2H). ^13^C-NMR (CDCl_3_, 100 MHz, 25 °C): δ 172.3, 159.1, 156.6, 153.2, 144.3, 137.3, 137.2, 135.1, 135.0, 130.33, 130.28, 129.50, 129.48, 128.6, 123.9, 115.8, 115.6, 51.8, 51.2, 45.9, 45.3, 35.9, 34.3, 32.63, 32.60, 31.5, 30.4, 29.4, 29.2, 12.6, 12.3, 7.74, 7.67, 7.57. ^19^F-NMR (CDCl_3_, 376 MHz, 25 °C): δ-119.7 (br s, 1F). HRMS (ESI+) calculated for C_24_H_24_ClFN_4_O_2_: 454.1572. Found: 454.1470. [α]^20^_D_ = −8.74 ± 0.16° (CH_2_Cl_2_).

### 4.2. Radiosynthesis

[^11^C]CO_2_ (11–22 GBq) was produced by a ^14^N(p,α)^11^C reaction using a TR19 cyclotron (Advanced Cyclotron Systems, Inc). The [^11^C]CO_2_ was subsequently converted to [^11^C]CH_3_I using a TracerLab FX_C_ Pro unit (GE Healthcare, Chicago, IL, USA). The transformation was typically accomplished within 10 min and resulted in a starting activity of 5.5–14.8 GBq [^11^C]CH_3_I.

All solvents and reagents were purchased from Sigma Aldrich and were used without further purification unless otherwise noted.

The chemical and radiochemical purity of the final product was determined by analytical HPLC using a ProStar HPLC (Varian, Palo Alto, CA, USA) with a Luna C18(2) column (5 µm, 250 × 4.6 mm^2^, 100 Å) (Phenomenex, Torrance, CA, USA) and a 50:50 0.3 M NH_4_COOH (pH 4.2):MeCN mobile phase at a flow rate of 1.5 mL/min. The HPLC was coupled to single wavelength UV-Vis detector and an Eckert & Ziegler FlowCount NaI(Tl) radio-HPLC scintillation detector (Bioscan, Washington DC, USA). Absorbance was measured at 254 nm. The retention time, t_R_, of the radiolabeled product was compared to the t_R_ of a non-radioactive standard.

#### 4.2.1. (*S*)-4-(4-(benzofuran-6-yl)phenyl)-5-((1-(cyclopropanecarbonyl)pyrrolidin-3-yl)methyl)-2-[methyl-^11^C]-2,4-dihydro-3H-1,2,4-triazol-3-one ([^11^C]**4**)

The [^11^C]CH_3_I was trapped on ascarite and released as a bolus into a capped reaction vial containing a pre-mixed solution of either 1 mg GSK2194069 and 3.0 µL 2.5 M NaOH in 350 µL DMSO or 1 mg (2.3 µmol) GSK2194069 and 5 mg (36 µmol) K_2_CO_3_ in 350 µL DMF. The reaction was stirred for 5 min at 80 °C before it was quenched by addition of 2 mL 60:40 0.3 M NH_4_COOH (pH 4.2):MeCN.

The quenched reaction mixture was purified using a Phenomenex Luna C18 column (5 µm, 250 × 10 mm^2^, 100 Å) and a 60:40 0.3 M NH_4_COOH (pH 4.2):MeCN mobile phase at a flow rate of 6 mL/min. The fraction containing the product was collected, diluted with H_2_O, and passed through a pre-conditioned Sep-Pak^®^ C18 Plus Light cartridge (Waters, Milford, MA, USA). The cartridge was washed with 10 mL H_2_O and dried under a flow of N_2_. [^11^C]**4** was eluted using 700 µL EtOH followed by 9.3 mL normal saline (0.9% NaCl). The purity of the final product was determined as described above.

#### 4.2.2. (*S*)-4-(4-(benzofuran-6-yl)-2-fluorophenyl)-5-((1-(cyclopropanecarbonyl)pyrrolidin-3-yl)methyl)-2-[methyl-^11^C]-2,4-dihydro-3H-1,2,4-triazol-3-one [^11^C]**5**

A solution of 1 mg (2.2 µmol) (*S*)-4-(4-(benzofuran-6-yl)-2-fluorophenyl)-5-((1-(cyclopropanecarbonyl)pyrrolidin-3-yl)methyl)-2,4-dihydro-3H-1,2,4-triazol-3-one (**2**) and 5.0 µL 2 M NaOH in 350 µL DMSO was stirred for 2 min at 80 °C. Then [^11^C]CH_3_I was introduced as a bolus into the sealed reaction vessel. The reaction was stirred for 5 min at 80 °C, then quenched by addition of 650 µL H_2_O + 0.01% TFA.

The quenched reaction product was purified using a Waters Bondapak^®^, C18 column (15–20 µm, 7.8 × 300 mm^2^, 125 Å) and an isocratic mobile phase of 50:50 H_2_O + 0.01 TFA/90% MeCN/H_2_O + 0.01% TFA at a flow rate of 4 mL/min. The retention time of the product was 10–13 min. The fraction corresponding to the product was collected and diluted to 30 mL with H_2_O. The diluted fraction was passed through a pre-conditioned Sep Pak^®^ C18 Plus Light cartridge (Waters). The cartridge was washed with 10 mL H_2_O and dried with air. [^11^C]**5** was eluted with 100 µL EtOH followed by 900 µL saline (0.9% NaCl). The purity of the final compound was determined as described above.

#### 4.2.3. (*S*)-4-(4′-chloro-3-fluoro-[1,1′-biphenyl]-4-yl)-5-((1-(cyclopropanecarbonyl)pyrrolidin-3-yl)methyl)-2-[methyl-^11^C]-2,4-dihydro-3H-1,2,4-triazol-3-one ([^11^C]**6**)

[^11^C]CH_3_I was introduced into a sealed reaction vial containing a premixed solution of 1 mg (2.3 µmol) (S)-4-(4′-chloro-3-fluoro-[1,1′-biphenyl]-4-yl)-5-((1-(cyclopropanecarbonyl)pyrrolidin-3-yl)methyl)-2,4-dihydro-3H-1,2,4-triazol-3-one (**3**) and 3.0 µL 2.5 M NaOH in 350 µL DMSO. The reaction was stirred for 5 min at 80 °C, then quenched with 600 µL 0.3 M NH_4_COOH (pH 4.2).

The quenched reaction mixture was purified using a Phenomenex Luna C18 column (5 µm, 250 × 10 mm^2^, 100 Å) and a 50:50 0.3 M NH_4_COOH (pH 4.2):MeCN mobile phase at a flow rate of 6 mL/min. The fraction containing the product was collected, diluted with H_2_O, and passed through a pre-conditioned Sep-Pak^®^ C18 Plus Light cartridge (Waters). The cartridge was washed with 10 mL H_2_O and dried under a flow of N_2_. [^11^C]**6** was eluted using 1 mL EtOH followed by 13 mL normal saline (0.9% NaCl). The purity of the final compound was determined as described above.

### 4.3. Cell Lines

The FASN-expressing human prostate cancer cell line LNCaP was obtained from the American Type Culture Collection (ATCC, Manassas, VA, USA). The low-expressing human prostate cancer cell line PC3 was also obtained from the ATCC. Cell culture supplies were purchased from Corning unless otherwise noted. Both cell lines were cultured in RPMI-1640 media supplemented with 10% heat-inactivated fetal bovine serum (FBS; GE Healthcare), 100 U/mL penicillin, 100 U/mL streptomycin, 1 mM sodium pyruvate (Gibco, ThermoFisher Scientific, Waltham, MA, USA), 0.45% glucose, MEM non-essential amino acids, and 2 mM glutamine (Gibco) at 37 °C and in 5% CO_2_. Cells were removed from flasks for passage or for transfer to 24-well assay plates by incubating them with 0.25% trypsin/ethylenediaminetetraacetic acid (EDTA).

### 4.4. Western Blotting

The LNCaP and PC3 cells were grown to confluence in RPMI-1640 media supplemented with 10% FBS, pen/strep, glutamine, pyruvate, glucose, and non-essential amino acids. The cells were lysed with 50 mM Tris.HCl (pH 7.4), 0.5 mM EDTA, and Pierce Halt™ Protease Inhibitor Cocktail (ThermoFisher) with 30–60 s grinding using Molecular Grinding Resin™ (Sigma Aldrich, St. Louis, MO, USA). Excised tumors were immediately flash frozen and stored at −80 °C. The samples were thawed and sliced, and the tissue slices were minced and then homogenized as described for the cell lines. A ratio of 4 parts lysis buffer to 1 part cellular material was maintained for all tumor samples. Cell protein content was determined using the BCA method (ThermoFisher) with bovine serum albumin as the standard. The blot was stained with either a rabbit polyclonal anti-FASN antibody from Bethyl Laboratories (1:1000, Bethyl Laboratories, Montgomery, TX, USA) or a mouse monoclonal anti-β-actin antibody (1:100, Santa Cruz Biotechnology, Dallas, TX, USA) followed by HRP-conjugated goat anti-mouse IgG (1:1000, Bio-Rad Laboratories, Hercules, CA, USA).

### 4.5. Binding to Prostate Cancer Cell Lines

LNCaP cells were seeded at a density of 2.5 × 10^5^ cells/mL in 24-well plates and grown for 24–48 h in RPMI-1640 media supplemented with 10% fetal bovine serum (FBS). The cells were a confluent monolayer at the time of the assay. Immediately prior to the assay, the growth medium was removed from each well under suction and replaced with 480 µL fresh RPMI-1640 media supplemented with 0.05% bovine serum albumin (BSA). A stock solution was prepared by adding 111–222 MBq of the ^11^C-labeled tracer in 1 mL 10% EtOH/saline to 25 mL of the RPMI-1640 media. From this stock solution were added 20 µL aliquots, containing approximately 111 kBq at time of addition, to each well. The experiment was performed in triplicate. The plates were incubated at 37 °C for 5, 10, 15, 20, 30, 40, or 50 min. Then the media was removed under suction, the wells were washed three times with PBS, and the cells were detached with 0.8 mL RIPA buffer. Non-specific binding was determined by immediately removing the media after addition to the wells. The cells were transferred to tubes and counted on a Wizard ^2^ automated y-counter (Perkin Elmer, Waltham, MA, USA). A 10% added activity standard was counted for quantification. Counts were corrected for decay, the background and non-specific binding were subtracted. The resulting values were expressed as a percentage of added activity ± standard error of the mean (SEM) and normalized to the protein content of each corresponding well.

PC3 cells were seeded at a density of 5 × 10^4^ cells/mL in 24-well plates and grown for 24–48 h in RPMI-media supplemented with 10% FBS. The cells were a confluent monolayer at the time of the assay. The binding was determined as described above. The resulting values were expressed as a percentage of added activity ± SEM and normalized to the protein content of each corresponding well.

The protein content of each sample was determined by analysis of a 5 µL aliquot of the harvested cell mixture after activity decayed to background.

### 4.6. Growth Inhibition Assay

The growth inhibition of LNCaP and PC3 following treatment with FASN inhibitors was carried out according to recently published methods [64]. Briefly, LNCaP and PC3 cells were seeded at 1 mL/well on 24-well plates at a density of 1 × 10^6^ cells/mL or 1.25 × 10^4^ cells/mL, respectively. The cells were seeded in RPMI-1640 media supplemented with 1% heat-inactivated FBS, 100 U/mL penicillin, 100 U/mL streptomycin, 1 mM sodium pyruvate, 0.45% glucose, MEM non-essential amino acids, and 2 mM glutamine. One day after seeding, when the cells had grown to approximately 50% confluence, the seeding media was removed and replaced with 2 mL of the culture media described above containing either 0.1% *v*/*v* DMSO (vehicle) or solutions of GSK2194069, **4**, **5**, **6** in DMSO. The concentration of DMSO in the culture media was 0.1% *v*/*v* for all inhibitor concentrations. The final concentration of inhibitor in the well ranged from 10^−4^ M to 10^−9^ M. The experiments were performed in triplicate. The treated cells were cultured for four days at 37 °C and in 5% CO_2_. Cell viability was then assessed by replacing the treatment media with 0.25 mM of a solution of Presto Blue (ThermoFisher), prepared as recommended by the supplier, and incubation for a further 40 min at 37 °C and in 5% CO_2_. After incubation, 0.2 mL was removed from the wells for quantification at 660_ex_ and 690_em_ nm. The resulting data was compared to the values obtained using the vehicle and expressed as the percentage of control viability. The results were analyzed and plotted as mean ± SEM using GraphPad Prism 8.4.3.

### 4.7. Animal Models

Animals were housed under standard conditions in approved facilities with 12 h light–dark cycles. Food and water were provided ad libitum throughout the course of the studies. Eight-week-old male inbred athymic nu/nu mice were purchased from The Jackson Laboratory. LNCaP cells were suspended at 4 × 10^7^ cells/mL in a 1:1 mixture of phosphate-buffered saline:Matrigel (BD Biosciences, San Jose, CA, USA). Each mouse was injected in the left flank with 0.25 mL of the cell suspension. Imaging studies were performed once tumors reached a volume of 150–500 mm^3^, approximately 4 weeks post inoculation.

### 4.8. MicroPET/CT Imaging

*Method A*: Tumor-bearing mice were injected intravenously with 100 µL of the final product solution, containing 8–10 MBq [^11^C]**4**, 9–11 MBq [^11^C]**5**, or 9–11 MBq [^11^C]**6**. The mice were then placed on the imaging bed and a 60 min dynamic acquisition was performed by microPET/CT (Siemens Inveon™, Siemens Medical Solutions USA, Malvern, PA, USA). The acquisition typically began 15 min after injection of the radiotracer. A CT scan was performed immediately upon conclusion of the microPET scan for attenuation correction and anatomical co-registration. The images were processed using open-source image processing software (AMIDE).

*Method B*: A 500 µL aliquot containing approximately 111 MBq (38 ng) [^11^C]**5** in 10% EtOH/saline was added to a vial containing 50 mg Captisol^®^ in 0.5 mL saline, prepared 4 h previously. The mixture was shaken vigorously for 10 min. A control solution was prepared by diluting a 500 µL aliquot containing approximately 111 MBq (38 ng) [^11^C]**5** in 10% EtOH/saline with 0.5 mL saline.

Two male athymic nu/nu mice bearing LNCaP xenograft tumors (150–500 mm^3^) were injected intravenously with 100 µL of the solution, containing 9–11 MBq [^11^C]**5** and 5 mg Captisol^®^. In parallel, two mice were injected intravenously with 100 µL of the control solution, containing 9–11 MBq. The mice were then placed on the imaging bed and a 60 min dynamic acquisition was performed by microPET/CT (Siemens Inveon™). The acquisition typically began 15 min after injection of [^11^C]**5**. A CT scan was performed immediately upon conclusion of the microPET scan for attenuation correction and anatomical co-registration. The images were processed using AMIDE.

### 4.9. Statistics

Statistical comparisons were drawn using the Tukey’s multiple comparison test (GraphPad Prism) with a significance level of *p* < 0.05.

## 5. Conclusions

Small molecule triazolones structurally related to GSK2194069, a potent FASN inhibitor, are readily ^11^C-methylated in good yield and high purity and molar activity under mild conditions. Of these compounds, [^11^C]**5** shows considerable promise in vitro as it reduces the viability of LNCaP cells with an EC_50_ comparable to GSK2194069 and is taken up to a significantly higher extent in FASN-positive LNCaP cells than in FASN-negative PC3 cells in vitro. However, the probes demonstrate remarkably poor tumor uptake in vivo, with the majority of the activity retained in lymph nodes and the hepatobiliary system. Future studies should target structural modifications that improve the pharmacokinetics of these triazolone FASN inhibitors.

## Data Availability

Raw data is available from the authors upon request.

## Supplementary Materials

The following supporting information can be downloaded at: online, Full experimental details, including Scheme S1: Synthesis of 4 from GSK2194069, and Scheme S2: Synthesis of non-radioactive standards 5 and 6 and their precursors 2 and 3; Figure S1: Radio (top) and UV (bottom) chromatograms of purified [^11^C]4; Figure S2: Radio (top) and UV (bottom) chromatograms of purified [^11^C]5; Figure S3: Radio (top) and UV (bottom) chromatograms of purified [^11^C]6; Figure S4. Analysis of FASN expression in cell lines and xenograft tumors by Western blot; Figure S5. Comparison of viability of LNCaP and PC3 cells treated with A. GSK2194069 or B. 2 (right); Figure S6. Comparison of [^11^C]5 binding to LNCaP cells cultured for >48 h (black) and <48 h (blue); and ^1^H-NMR, ^13^C-NMR, and ^19^F-NMR spectra of all final compounds and intermediates.

## Sample Availability

Samples of compounds **2**–**6** manuscript are available from the authors upon request.
