# Supplementary material for: Synthesis and Evaluation of 11C-Labeled Triazolones as Probes for Imaging Fatty Acid Synthase Expression by Positron Emission Tomography
Source: Molecules. 2022 Feb 25;27(5):1552. doi: 10.3390/molecules27051552 (PMC8911806; doi:10.3390/molecules27051552)
Supplement: Supplementary file 1 [file molecules-27-01552-s001.zip › molecules-1570505-supplementary.pdf]

# Supplementary Materials

**Synthesis and Evaluation of $^{11}$C-Labeled Triazolones as Probes for Imaging Fatty Acid Synthase Expression by Positron Emission Tomography**

James M. Kelly*, Thomas M. Jeitner, Nicole Waterhouse, Wenchao Qu, Ethan J. Linstad, Banafshe Samani, Clarence Williams, Jr., Anastasia Nikolopoulou, Alejandro Amor-Coarasa, Stephen G. DiMagno, John W. Babich

* Corresponding author:

James M. Kelly, Ph.D.
Department of Radiology
Weill Cornell Medicine
Belfer Research Building
Room 1604
413 E 69th St
New York, NY 10021
jak2046@med.cornell.edu

**Table of Contents**

1. Synthesis of Precursor Compounds and Non-Radioactive Standards……………………………………………………2

2. UV and Radiochromatograms of Purified Products……………………………………………………………………………9

3. Determination of FASN Expression in LNCaP and PC3 Cells…………………………………………………………….12

4. Growth Inhibition Assay…………………………………………………………………………………………………………….13

5. Comparison of *In vitro* Uptake in Aggregated versus Sub-Confluent LNCaP Cells………………………………14

6. $^{1}$H and $^{13}$C NMR Spectra of Compounds……………………………………………………………………………………….15

## 1. Synthesis of Precursor Compounds and Non-Radioactive Standards

**General methods:** All materials were obtained from commercial sources and used as received unless otherwise noted. Acetonitrile and $d_3$-acetonitrile (Cambridge Isotope Laboratories) were stirred over $P_2O_5$ (12 h), distilled into flame-dried storage tubes, and transferred into an inert atmosphere glove box. All glassware and NMR tubes were oven dried (140 °C) for 24 h before they were transferred into the glove box for use. Glove box manipulations were performed under nitrogen in an MBraun Labmaster 130, equipped with a recirculating purifier, which removed oxygen and water. All NMR experiments reported were performed using a Bruker Avance 300 MHz, 400 MHz, 500 MHz, 600 MHz, or 700 MHz NMR spectrometers in the NMR laboratory at the University of Nebraska, the University of Illinois at Chicago, or Weill Cornell Medicine, and calibrated using residual protonated solvent. Yields from NMR scale reactions were determined by using the residual solvent peak as an internal standard. All *J* coupling values are given in Hz. Masses were determined by high resolution LC-MS or HR-MS using electrospray ionization (ESI). LC-MS mass determinations were performed on a Waters ACQUITY UPLC® coupled to a Waters ZSpray™ ionizer and a Waters SQ Detector 2. The column used for the chromatography component was a Phenomenex Kinetex C18, 50 x 2.1 mm, 1.7 µm and the mobile phase was a gradient of 5-95% MeCN + 0.1% TFA in $H_2O$ + 0.1% TFA over 5 min. HR-MS mass determinations were performed on a Shimadzu LCMS IT-TOF™. The chromatography unit was bypassed for mass measurements. The optical rotation was determined using a JASCO P-1020 polarimeter. The samples were dissolved in $CH_2Cl_2$ to a concentration of 0.3% *w/v* and analyses were performed at 589 nm and 20 °C. Optical rotations are reported as the mean of 10 replicates ± standard deviation.

*Synthesis of* ***4****:*

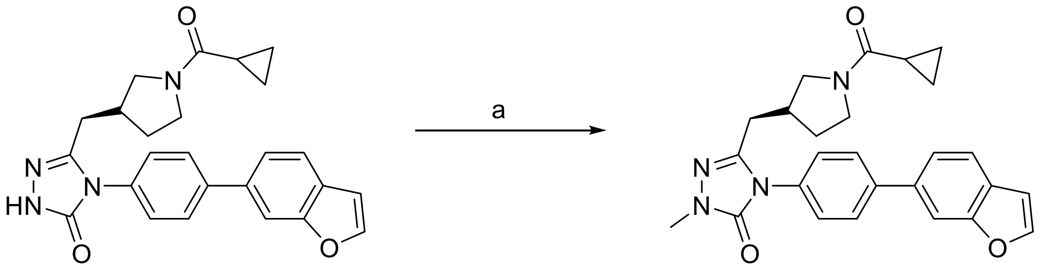


(**1**) (**4**)

**Scheme S1.** Synthesis of **4** from GSK2194069. a. $K_2CO_3$, $CH_3I$, DMF.

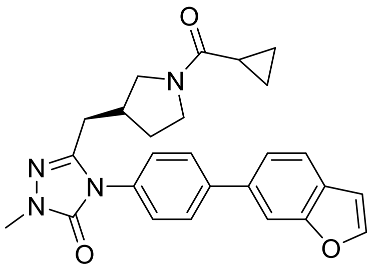

**(*S*)-4-(4-(Benzofuran-6-yl)phenyl)-5-((1-(cyclopropanecarbonyl)pyrrolidin-3-yl)methyl)-2-methyl-2,4-dihydro-3*H*-1,2,4-triazol-3-one (*4*):** To a solution of 3 mg (7 µmol) GSK2194069 in 0.5 mL DMF was added 6.4 mg (46 µmol) $K_2CO_3$ and 10 µL (160 µmol) methyl iodide. The reaction was stirred at room temperature for 24 h. The reaction was quenched with 2 mL 58% *v/v* 0.3 M $NH_4COOH$/MeCN, and the reaction was

purified in two fractions by semi-prep HPLC using a Phenomenex Luna C18(2) column, 250x10 mm and monitoring UV absorption at 254 nm. The mobile phase was 58% *v/v* 0.3 M $NH_4COOH$/MeCN, and the flow rate was 5 mL/min. Under these conditions, the product eluted with a retention time of 11.2 min. The fractions containing the product was collected, combined, and diluted with 200 mL $H_2O$. The diluted sample was passed through a pre-conditioned Sep-Pak C18 Plus Light cartridge (Waters). The cartridge was washed with 20 mL $H_2O$ and eluted with 1 mL EtOH and 1 mL MeCN. The organic solvent was removed by heating to 75 °C under a stream of $N_2$ to give **4** as a white powder (2.8 mg, 90%). $^1$H NMR ($CDCl_3$, 500 MHz, 25 °C): δ 7.83 (d, *J* = 1.5, 1H), 7.76 (d, *J* = 8.5, 2H), 7.71 (d, *J* = 2.5, 1H), 7.61 (d, *J* = 8.5, 1H), 7.55 (dd, *J* = 8.5, 2.0, 1H), 7.38 (d, *J* = 8.0, 2H), 6.86 (d, J = 2.0, 0.5, 1H), 3.85 (m, 1H), 3.70 (m, 1H), 3.53 (m, 1H), 3.18 (m, 1H), 3.55 (s, 3H), 2.64-2.42 (br m, 2.5H), 2.26-2.15 (br m, 1H), 2.10-2.02 (m, 0.5H), 1.77-1.68 (m, 0.5H), 1.61-1.56 (m, 1.5H), 1.02-0.96 (m, 2H), 0.79-0.74 (m, 2H). $^{13}$C NMR ($CDCl_3$, 150 MHz, 25 °C): δ 172.3, 154.8, 153.5, 145.9, 142.8, 137.2, 135.1, 131.5, 128.9, 128.8, 128.1, 127.4, 127.3, 123.9, 119.9, 111.8, 106.8, 51.8, 45.3, 42.8, 32.5, 29.7, 22.8, 12.2, 7.6, 7.5. LCMS calc. for $C_{26}H_{26}N_4O_3$ $[M+H]^+$: 443.20. Found: 443.24. $[\alpha]^{20}_D$ = -8.04 ± 0.59° ($CH_2Cl_2$).

*Synthesis of* ***5*** *and* ***6****:*

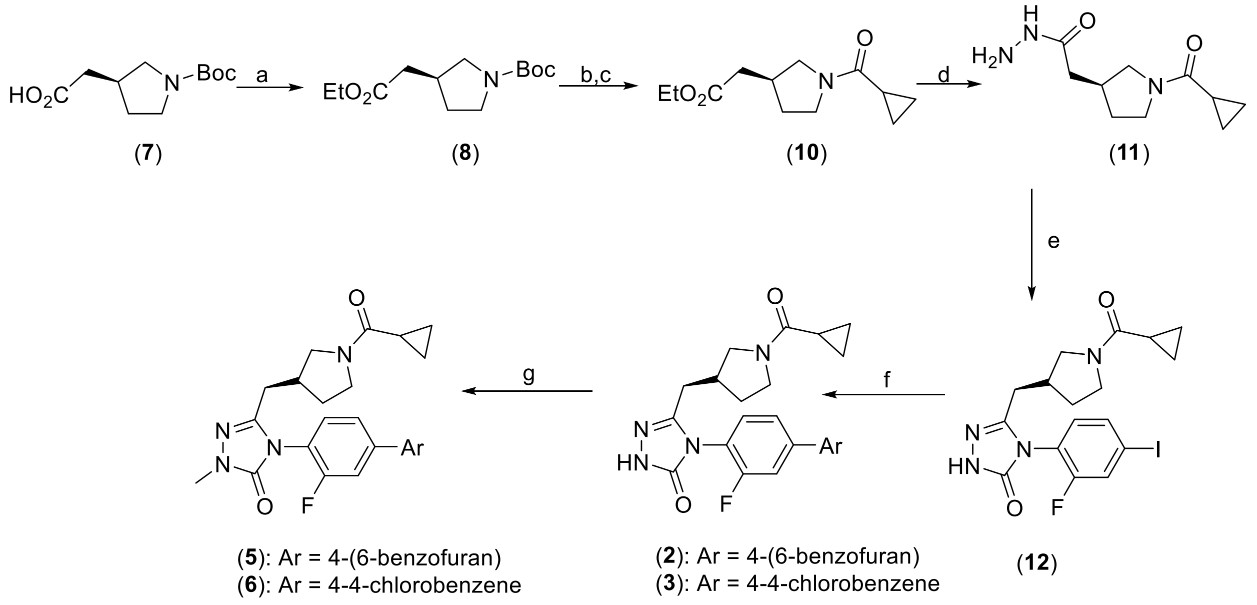


**Scheme S2**. Synthesis of non-radioactive standards **5** and **6** and their precursors **2** and **3**. a. EDC•HCl, DMAP, EtOH, $CH_2Cl_2$; b. HCl/dioxane; c. $C_3H_5COCl$, DIPEA, $CH_2Cl_2$; d. $NH_2NH_2$•$H_2O$, EtOH; e. 2-fluoro-4-iodoaniline, $NEt_3$, triphosgene, THF; f. $ArB(OH)_2$, $Na_2CO_3$, dioxane/$H_2O$, $PdCl_2$(dppf), *n*-BuLi, THF; .MeI, $K_2CO_3$, DMF.

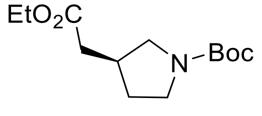


***tert*-Butyl (*S*)-3-(2-ethoxy-2-oxoethyl)pyrrolidine-1-carboxylate (8):** (S)-2-(1-(*tert*-Butoxycarbonyl) pyrrolidin-3-yl)acetic acid (**7**) (8 g, 35 mmol) was dissolved in dichloromethane (70 mL). Then EDC•HCl (7.4 g, 38.6 mmol), DMAP (0.4 g, 3.3 mmol), and EtOH (4.5 mL, 77 mmol) were added successively. The reaction was stirred for 16 h at room temperature. Then it was diluted with dichloromethane (70 mL), washed with

1 M HCl (10 mL) and saturated aqueous sodium carbonate (10 mL), dried over sodium sulfate, filtered, and concentrated under reduced pressure to give **8** as a pale yellow oil (9 g, 99%), which was used without further purification. $^1$H NMR ($CDCl_3$, 400 MHz, 25 °C): δ 4.14 (br q, J = 7.1, 2H), 3.62-3.55 (m, 1H), 3.49-3.39 (m, 1H), 3.33-3.23 (m, 1H), 2.99-2.89 (m, 1H), 2.55 (sept J = 7.4, 1H), 2.42-2.31 (m, 2H), 2.06 (br s, 1H), 1.58-1.50 (m, 1H), 1.45 (s, 9H), 1.25 (t, J = 7.1, 3H)

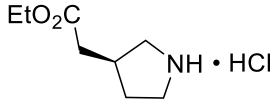


**Ethyl (*S*)-2-(pyrrolidin-3-yl)acetate hydrochloride (9):** In a round bottom flask, pyrrolidine **8** (9 g, 35 mmol) was dissolved in 4 M HCl in dioxane (46 mL, 184 mmol), with evolution of gas, and stirred for 5 h at room temperature. The solvent was removed under high vacuum to give **9** as an off white solid (6.7 g, 99%) which was used without further purification. $^1$H NMR ($CDCl_3$, 400 MHz, 25 °C): δ 9.80 (br s, 2H), 4.13 (q, J = 7.1, 2H), 3.62-3.55 (m, 1H), 3.49-3.39 (m, 1H), 3.33-3.23 (m, 1H), 3.02-2.93 (m, 1H), 2.72 (sept, J = 7.7, 1H), 2.50 (d, J = 7.2, 2H), 2.30-2.24 (m, 1H), 1.76-1.68 (m, 1H), 1.25 (t, J = 7.1, 3H).

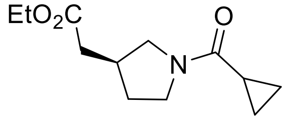


**Ethyl (*S*)-2-(1-(cyclopropanecarbonyl)pyrrolidin-3-yl)acetate (10):** Pyrrolidine **9** (6.5 g, 33.6 mmol) was dissolved in dichloromethane (65 mL) under nitrogen with stirring. DIPEA (11 mL, 66 mmol) was added slowly and the solution cooled in an ice bath. Cyclopropylcarbonyl chloride (3.7 mL, 40.1 mmol) was added slowly and the reaction allowed to warm to room temperature with stirring for 2 h. Then the reaction was poured into $H_2O$ (50 mL), the organic layer separated, and the aqueous phase extracted with DCM (10 mL x 3). The organic layers were combined and washed with sat. $NaHCO_3$ (10 mL), dried over sodium sulfate and concentrated under reduced pressure. The resulting oil was placed under high vacuum for 1 d to yield **10** as an amber oil (7.46 g, 98%), which was used without further purification. $^1$H NMR ($CDCl_3$, 400 MHz, 25 °C): δ 4.15 (q, J = 7.1, 1H), 4.13 (q, J = 7.1, 1H), 3.95-3.90 (m, 0.5H), 3.79-3.72 (m, 1H), 3.67-3.57 (m, 1H), 3.41-3.34 (m, 0.5H), 3.27-3.22 (m, 0.5H), 3.08-3.03 (m, 0.5H), 2.68 (sept, J = 7.6, 0.5H), 2.58 (sept, J = 7.6, 0.5H), 2.51-2.33 (m, 2H), 2.25-2.18 (m, 0.5H), 2.12-2.05 (m, 0.5H), 1.74-1.65 (m, 0.5H), 1.62-1.54 (m, 1.5H), 1.27 (t, J = 6.8, 1.5H), 1.25 (t, J = 6.8, 1.5H), 1.02-0.93 (m, 2H), 0.78-0.69 (m, 2H).

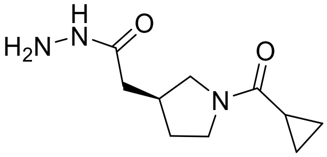


**(*S*)-2-(1-(Cyclopropanecarbonyl)pyrrolidin-3-yl)acetohydrazide (11):** Ester **10** (6.25 g, 27.7 mmol) and hydrazine hydrate (36 mL, 550 mmol) were dissolved in EtOH (60 mL) and refluxed for 16 h. The solvent was removed, $H_2O$ azeotropically removed with isopropanol, and the resulting oil placed under high vacuum for 3 d to give **11** as a sticky, light amber oil (5.5 g, 94%). The compound was stored in the dark until further use. $^1$H NMR ($CDCl_3$, 400 MHz, 25 °C): δ 6.80 (br s, 1H), 3.94-3.85 (br m, 2.5H), 3.78-3.71 (m, 1H), 3.66-3.58 (m, 1H), 3.44-3.36 (m, 0.5H), 3.31-3.27 (m, 0.5H), 3.09-3.04 (m, 0.5H), 2.74 (sept, J = 7.3, 0.5H), 2.64 (sept, J = 7.5, 0.5H), 2.31-2.20 (m, 2H) 2.12-2.05 (m, 0.5H), 1.77-1.67 (m, 0.5H), 1.66-1.52 (m,

2H), 1.03-0.92 (m, 2H), 0.80-0.71 (m, 2H). $^{13}C$ NMR ($CDCl_3$, 100 MHz, 25 °C): δ 172.3, 171.9, 152.1, 51.8, 51.0, 46.0, 45.2, 39.2, 37.3, 36.0, 35.2, 34.4, 31.7, 30.2, 27.5, 16.5, 12.5, 12.2, 7.6, 7.5, 7.4, 7.3. HRMS (ESI) calculated for $C_{11}H_{17}N_3O_2$: 211.1321. Found: 211.1297. $\Delta_m$ = 11.4 ppm.

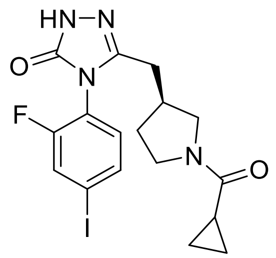


**(*S*)-5-((1-(Cyclopropanecarbonyl)pyrrolidin-3-yl)methyl)-4-(2-fluoro-4-iodophenyl)-2,4-dihydro-3*H*-1,2,4-triazol-3-one (12):** To a flame-dried Schlenk tube (solution A) was added a solution of 2-fluoro-4-iodoaniline (4.3 g, 18 mmol) in dry THF (90 mL). Dry triethylamine (20 mL, 144 mmol) was added, and the resulting mixture was stirred. To a separate flame-dried Schlenk flask (solution B), was added a stirred solution of triphosgene (5.4 g, 18 mmol) in dry THF (90 mL). Solution A was slowly added to solution B via cannula, and the resulting slurry was stirred for 16 h at room temperature. The reaction solution was filtered through an air-free filter funnel with a fine glass frit into a fresh, flame-dried Schlenk flask and the solvent removed under vacuum to give a red oil. Dry dichloromethane (60 mL) was added and the mixture was stirred. In a nitrogen atmosphere glove box, hydrazide **11** (1.9 g, 3.8 mmol) was dissolved in dry dichloromethane (25 mL) and added to the stirred reaction solution. The reaction was stirred for 16 h at room temperature, at which point a large amount of solid material (product) had precipitated. The product semi-carbazide was recovered by filtering the reaction solution through a 0.45 µm PTFE membrane filter to give a light amber solid (4.2 g, 94%). Then the semi-carbazide (1 g, 2.1 mmol) was suspended in 10% isopropanol/water (70 mL), $K_2CO_3$ (1.5 g, 10.5 mmol) added, and the mixture refluxed for 16 h. The solution was acidified to pH 5-5.5, extracted with ethyl acetate (10 mL x 5), dried over sodium sulfate, and concentrated under reduced pressure. The crude product was purified by column chromatography (100% EtOAc → 5% MeOH/EtOAc) to give **12** as a colorless solid (0.55 g, 57%). [Rotamers present in NMR spectra] $^1H$ NMR ($CDCl_3$, 400 MHz, 25 °C): δ 10.45 (br s, 0.5H), 10.40 (br s, 0.5H), 7.68-7.64 (m, 2H), 7.09 (dd, J = 8.2, 7.9, 1H), 3.93-3.89 (m, 0.5H), 3.76-3.68 (m, 1H), 3.64-3.55 (m, 1H), 3.41-3.34 (m, 0.5H), 3.26-3.22 (m, 0.5H), 3.07-3.02 (m, 0.5H), 2.68-2.59 (m, 0.5H), 2.55-2.40 (m, 2.5H), 2.22-2.19 (m, 0.5H), 2.09-2.01 (m, 0.5H), 1.73-1.63 (m, 0.5H), 1.59-1.49 (m, 1.5H), 1.05-0.92 (m, 2H), 0.80-0.69 (m, 2H). $^{13}C$ NMR ($CDCl_3$, 100 MHz, 25 °C): δ 172.42, 172.37, 158.5, 155.9, 154.75, 154.69, 146.0, 135.0, 131.2, 131.1, 126.9, 126.8, 126.7, 126.6, 120.3, 120.20, 120.18, 120.1, 95.4, 95.33, 95.29, 95.2, 77.4, 51.8, 51.1, 45.9, 45.3, 35.9, 34.2, 31.6, 30.4, 29.4, 29.3, 12.6, 12.4, 7.79, 7.73, 7.6. $^{19}F$ NMR ($CDCl_3$, 376 MHz, 25 °C): δ -117.7 (br s, 1F). HRMS (ESI) calculated for $C_{17}H_{18}FIN_4O_2$: 456.0458. Found: 456.0457. $\Delta_m$ = 0.2 ppm.

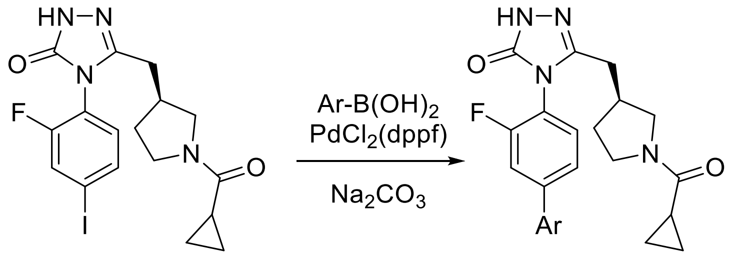


**General cross-coupling procedure:**

Triazolone **12** (100 mg, 0.22 mmol), arylboronic acid (0.26 mmol) and $Na_2CO_3$ (91 mg, 0.66 mmol) were weighed out in a nitrogen atmosphere glove box, transferred to a Schlenk tube, removed from the box and placed under nitrogen using a Schlenk line. Degassed 3:1 dioxane:water (1.5 mL) was added with stirring. In a nitrogen glove box, $PdCl_2$(dppf) (1 mg, cat.) was suspended in THF (0.5 mL), activated with a drop of *n*-BuLi (leading to a deep red solution), and added to the reaction mixture. The reaction vessels were sealed with a greased glass stopper and heated to 100 °C for 16 h. Then the reaction was acidified to pH 5-5.5 and extracted with ethyl acetate (2 mL x 4). The extracts were combined, dried over sodium sulfate, and concentrated under reduced pressure. The crude products were purified by column chromatography (typically 50% EtOAc/hexane → 10% MeOH/EtOAc) to give **2** and **3** as amber oils.

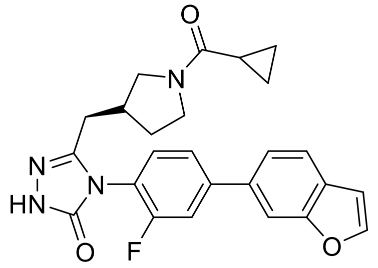

**(*R*)-4-(4-(Benzofuran-6-yl)-2-fluorophenyl)-5-((1-(cyclopropanecarbonyl)pyrrolidin-3-yl)methyl)-2,4-dihydro-3*H*-1,2,4-triazol-3-one (2):** (95 mg, 97%). $^1$H NMR ($CDCl_3$, 400 MHz, 25 °C): δ 10.24 (br s, 0.5H), 10.12 (br s, 0.5H), 7.80 (br s, 1H), 7.69 (br s, 1H), 7.60 (dd, J = 8.5, 2.8, 1H), 7.56-7.50 (m, 3H), 7.42 (dt, J = 11.7, 2.0, 1H), 6.84 (br s, 1H), 3.96-3.92 (m, 0.5H), 3.78-3.69 (m, 1H), 3.66-3.58 (m, 1H), 3.42-3.35 (m, 0.5H), 3.30-3.26 (m, 0.5H), 3.10-3.06 (m, 0.5H), 2.74-2.64 (m, 0.5H), 2.64-2.42 (br m, 2.5H), 2.26-2.15 (br m, 0.5H), 2.10-2.02 (m, 0.5H), 1.77-1.68 (m, 0.5H), 1.62-1.54 (m, 1.5H), 1.02-0.93 (m, 2H), 0.78-0.69 (m, 2H). $^{13}$C NMR ($CDCl_3$, 100 MHz, 25 °C): δ 172.4, 159.1, 156.6, 155.3, 155.2, 155.1, 146.6, 146.3, 146.2, 146.0, 145.93, 145.90, 145.8, 134.02, 133.95, 130.1, 130.0, 128.39, 128.37, 124.4, 123.9, 120.1, 118.6, 118.52, 118.48, 118.39, 116.1, 115.9, 112.13, 112.09, 106.9, 51.8, 51.2, 51.0, 45.9, 45.3, 36.0, 34.3, 31.6, 30.5, 29.5, 29.3, 12.6, 12.4, 7.8, 7.7, 7.6. $^{19}$F NMR ($CDCl_3$, 376 MHz, 25 °C): δ -120.2 (br s, 1F). HRMS (ESI) calculated for $C_{25}H_{23}FN_4O_3$: 446.1754. Found: 446.1685. $\Delta_m$ = 15.4 ppm. $[\alpha]^{20}_D$ = -8.35 ± 0.30° ($CH_2Cl_2$).

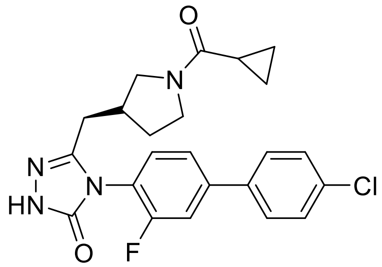

**(*R*)-4-(4'-Chloro-3-fluoro-[1,1'-biphenyl]-4-yl)-5-((1-(cyclopropanecarbonyl)pyrrolidin-3-yl)methyl)-2,4-dihydro-3H-1,2,4-triazol-3-one (3):** (95 mg, 98%). $^1$H NMR ($CDCl_3$, 400 MHz, 25 °C): δ 10.94 (br s, 1H), 7.50 (dd, J = 8.5, 1.6, 2H), 7.47-7.39 (m, 5H), 3.93-3.89 (m, 0.5H), 3.75-3.67 (m, 1H), 3.63-3.53 (m, 1H), 3.40-3.33 (m, 0.5H), 3.27-3.23 (m, 0.5H), 3.07-3.02 (m, 0.5H), 2.70-2.63 (m, 0.5H), 2.63-2.40 (m, 2.5H), 2.23-2.14 (m, 0.5H), 2.08-2.00 (m, 0.5H), 1.73-1.64 (m, 0.5H), 1.59-1.49 (m, 1.5H), 1.01-0.91 (m, 2H), 0.78-0.69 (m, 2H). $^{13}$C NMR ($CDCl_3$, 100 MHz, 25 °C): δ 172.5, 159.1, 156.5, 155.15, 155.09, 146.2, 144.1, 144.0, 143.9, 137.2, 137.1, 135.0, 134.9, 130.3, 130.2, 129.41, 129.38, 128.5, 123.9, 119.31, 119.23, 119.18, 119.10, 115.7, 115.5, 51.8, 51.2, 45.9, 45.3, 35.9, 34.3, 31.5, 30.4, 29.4, 29.2, 12.6, 12.3, 7.74, 7.67, 7.57.

$^{19}$F NMR ($CDCl_3$, 376 MHz, 25 °C): δ -119.7 (br s, 1F). HRMS (ESI) calculated for $C_{23}H_{22}ClFN_4O_2$: 440.1415. Found: 440.1339. $\Delta_m$ = 17.3 ppm. $[\alpha]^{20}_D$ = -9.37 ± 0.34° ($CH_2Cl_2$).

*Synthesis of **5** and **6**:*

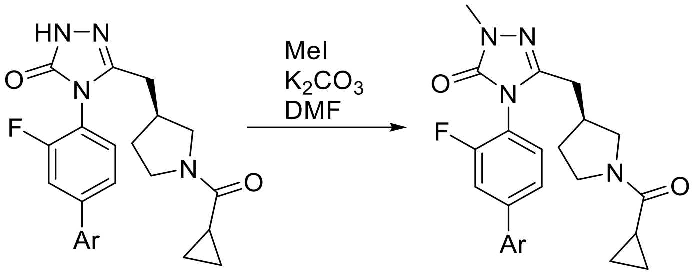


**General triazolone methylation procedure**:

Triazolone **2** or **3** (0.034 mmol) and $K_2CO_3$ (0.07 mmol) were combined in a storage tube with dry DMF (0.5 mL). Methyl iodide (3 µL, 0.048 mmol) was added, the tube was sealed, and the reaction was heated to 80 °C for 16 h. Then the reaction was poured into $H_2O$ (5 mL) and extracted with ethyl acetate (2 mL x 3). The organic extracts were combined, washed successively with $H_2O$ (2 mL) and brine (2 mL), dried over sodium sulfate, and concentrated under reduced pressure to afford *N*-methyltriazolones **5** and **6**.

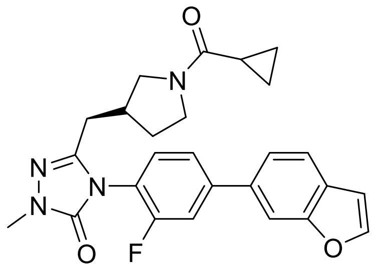

**(*R*)-4-(4-(Benzofuran-6-yl)-2-fluorophenyl)-5-((1-(cyclopropanecarbonyl)pyrrolidin-3-yl)methyl)-2-methyl-2,4-dihydro-3*H*-1,2,4-triazol-3-one (5):** (12 mg, 78%). $^1$H NMR ($CDCl_3$, 400 MHz, 25 °C): δ 7.80 (s, 1H), 7.69 (s, 1H), 7.59 (d, J = 8.4, 1H), 7.54-7.48 (m, 3H), 7.39 (dd, J = 8.7, 7.7, 1H), 6.84 (s, 1H), 3.94-3.90 (m, 0.5H), 3.79-3.69 (m, 1H), 3.65-3.56 (m, 1H), 3.52 (d, J = 6.9, 3H), 3.42-3.35 (m, 0.5H), 3.28-3.24 (m, 0.5H), 3.08-3.04 (m, 0.5H), 2.72-2.63 (m, 0.5H), 2.63-2.45 (br m, 2.5H), 2.26-2.15 (br m, 0.5H), 2.10-2.02 (m, 0.5H), 1.75-1.66 (m, 0.5H), 1.60-1.51 (m, 1.5H), 1.05-0.92 (m, 2H), 0.78-0.69 (m, 2H). $^{13}$C NMR ($CDCl_3$, 100 MHz, 25 °C): δ 172.3, 159.1, 156.6, 155.2, 146.24, 146.20, 145.84, 145.76, 145.73, 145.66, 144.5, 134.03, 133.98, 130.1, 130.0, 128.4, 124.3, 123.9, 120.1, 119.1, 119.02, 118.95, 118.89, 116.0, 115.9, 112.09, 112.07, 106.9, 51.8, 51.2, 51.0, 45.9, 45.3, 36.0, 34.3, 32.7, 31.6, 30.5, 29.5, 29.3, 12.6, 12.4, 7.8, 7.7, 7.6. $^{19}$F NMR ($CDCl_3$, 376 MHz, 25 °C): δ -120.3 (br s, 1F). HRMS (ESI) calculated for $C_{26}H_{25}FN_4O_3$: 460.1911. Found: 460.1846. $\Delta_m$ = 14.1 ppm. $[\alpha]^{20}_D$ = -2.99 ± 0.28° ($CH_2Cl_2$).

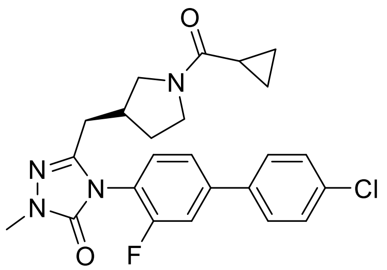

**(*R*)-4-(4'-Chloro-3-fluoro-[1,1'-biphenyl]-4-yl)-5-((1-(cyclopropanecarbonyl)pyrrolidin-3-yl)methyl)-2-methyl-2,4-dihydro-3*H*-1,2,4-triazol-3-one (6):** (15 mg, 60%). $^{1}$H NMR ($CDCl_3$, 400 MHz, 25 °C): δ 10.94 (br s, 1H), 7.52 (br d, J = 8.4, 2H), 7.47-7.39 (m, 5H), 3.93-3.89 (m, 0.5H), 3.75-3.67 (m, 1H), 3.63-3.56 (m, 1H), 3.52 (s, 1.5H), 3.51 (s, 1.5H), 3.40-3.33 (m, 0.5H), 3.27-3.23 (m, 0.5H), 3.07-3.02 (m, 0.5H), 2.70-2.63 (m, 0.5H), 2.63-2.40 (m, 2.5H), 2.23-2.14 (m, 0.5H), 2.08-2.00 (m, 0.5H), 1.73-1.64 (m, 0.5H), 1.59-1.49 (m, 1.5H), 1.01-0.91 (m, 2H), 0.78-0.69 (m, 2H). $^{13}$C NMR ($CDCl_3$, 100 MHz, 25 °C): δ 172.3, 159.1, 156.6, 153.2, 144.3, 137.3, 137.2, 135.1, 135.0, 130.33, 130.28, 129.50, 129.48, 128.6, 123.9, 115.8, 115.6, 51.8, 51.2, 45.9, 45.3, 35.9, 34.3, 32.63, 32.60, 31.5, 30.4, 29.4, 29.2, 12.6, 12.3, 7.74, 7.67, 7.57. $^{19}$F NMR ($CDCl_3$, 376 MHz, 25 °C): δ -119.7 (br s, 1F). HRMS (ESI) calculated for $C_{24}H_{24}ClFN_4O_2$: 454.1572. Found: 454.1470. $\Delta_m$ = 22.5 ppm. $[\alpha]^{20}_{D}$ = -8.74 ± 0.16° ($CH_2Cl_2$).

## 2. UV and Radiochromatograms of Purified Products

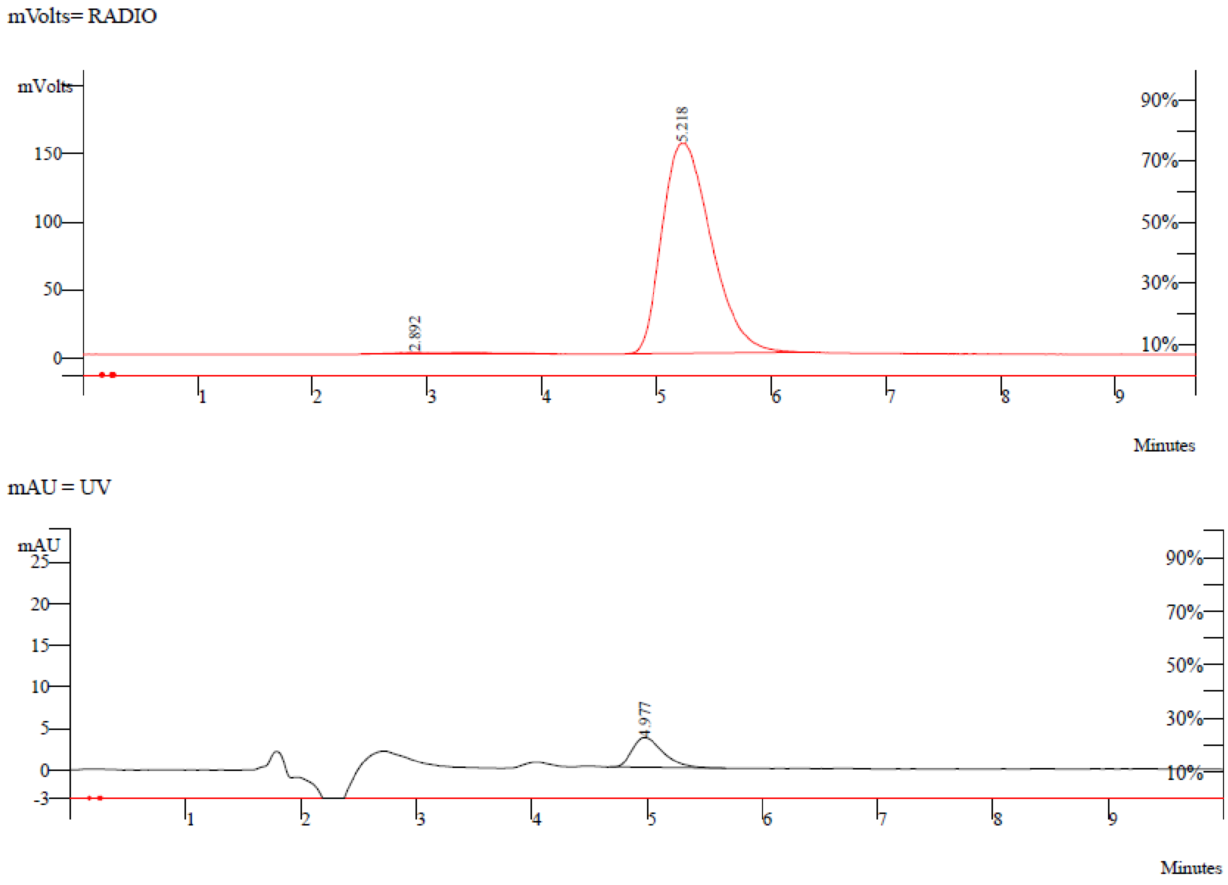


**Figure S1.** Radio (top) and UV (bottom) chromatograms of purified [$^{11}$C]**4**. Analysis was performed on a Phenomenex Luna C18(2) column (5 µm, 250 x 4.6 mm, 100 Å) using a 50:50 0.3 M $NH_4COOH$ (pH 4.2):MeCN mobile phase at a flow rate of 1.5 mL/min. The non-radioactive standard eluted with a retention time, $t_R$, of 4.94 min.

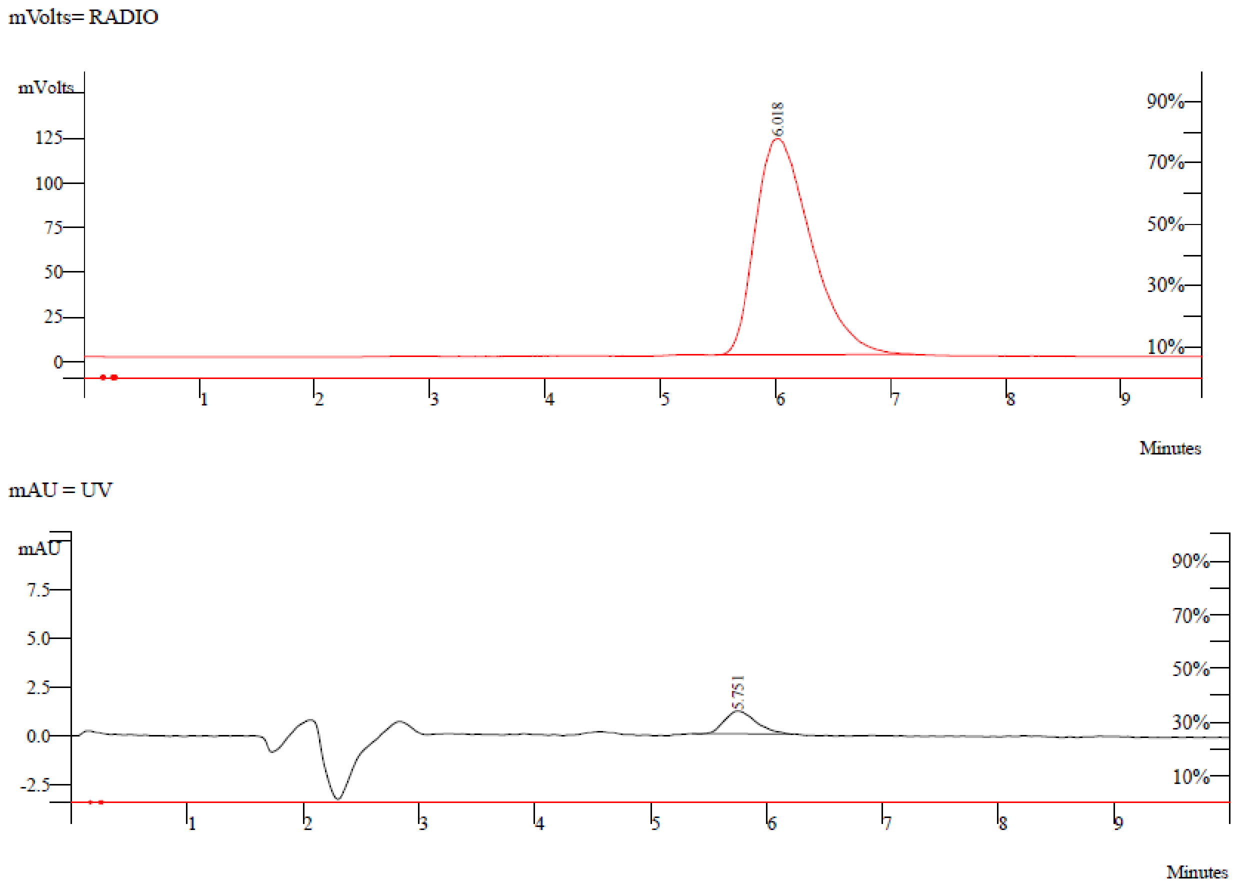


**Figure S2.** Radio (top) and UV (bottom) chromatograms of purified [$^{11}$C]**5**. Analysis was performed on a Phenomenex Luna C18(2) column (5 µm, 250 x 4.6 mm, 100 Å) using a 50:50 0.3 M $NH_4COOH$ (pH 4.2):MeCN mobile phase at a flow rate of 1.5 mL/min. The non-radioactive standard eluted with a retention time, $t_R$, of 5.77 min.

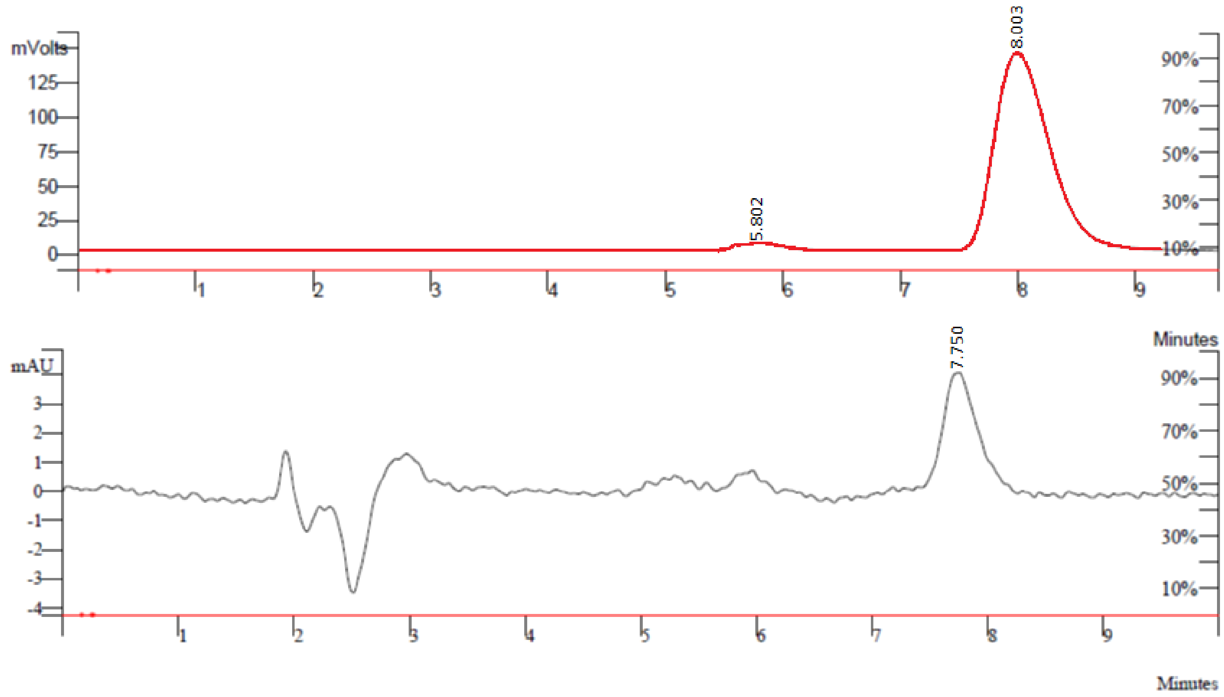


**Figure S3.** Radio (top) and UV (bottom) chromatograms of purified [$^{11}$C]**6**. Analysis was performed on a Phenomenex Luna C18(2) column (5 µm, 250 x 4.6 mm, 100 Å) using a 50:50 0.3 M $NH_4COOH$ (pH 4.2):MeCN mobile phase at a flow rate of 1.5 mL/min. The non-radioactive standard eluted with a retention time, $t_R$, of 7.69 min.

3. **Determination of FASN Expression in LNCaP and PC3 cells**

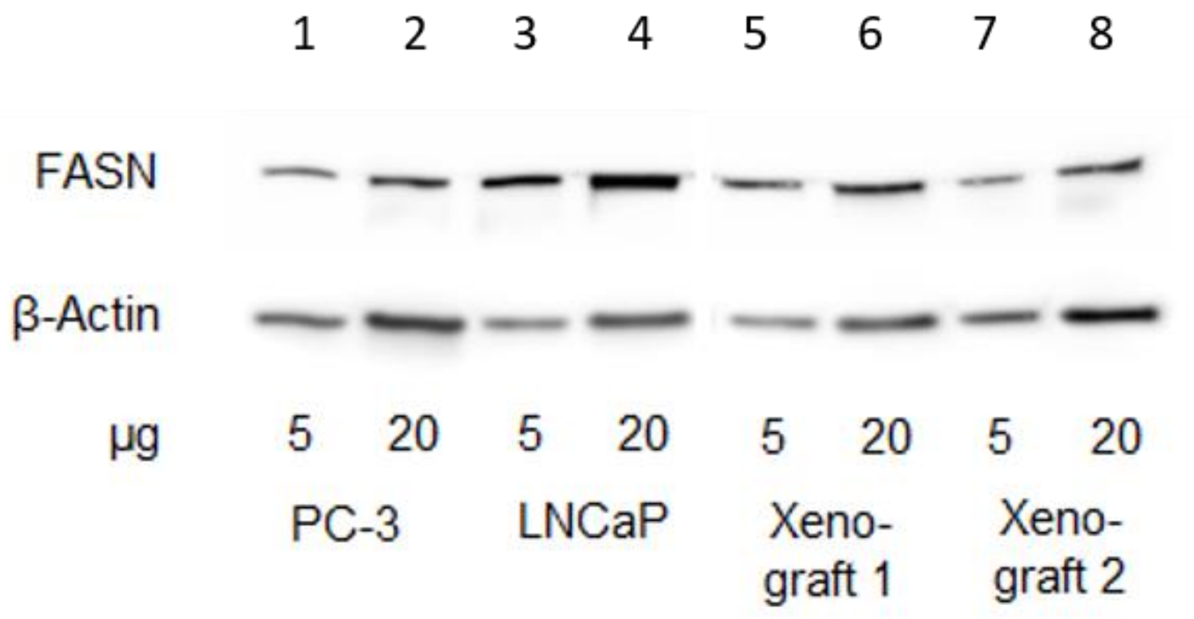


**Figure S4.** Shown are the FASN and β-actin staining due to 5 and 20 µg PC-3 (lanes 1 and 2), 5 and 20 µg LNCaP (lanes 3 and 4), 5 and 20 µg excised LNCaP xenograft tumor #1 (lanes 5 and 6), and 5 and 20 µg LNCaP xenograft tumor #2 (lanes 7 and 8).

## 4. Growth Inhibition Assay

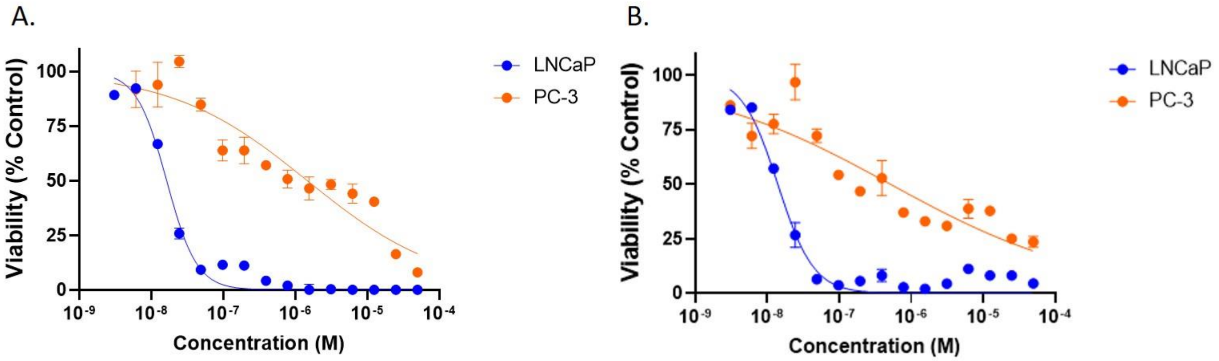


**Figure S5.** Comparison of viability of LNCaP and PC3 cells treated with **A.** GSK2194069 or **B. 2** (right).

## 5. Comparison of *In vitro* Uptake in Aggregated versus Sub-Confluence LNCaP cells

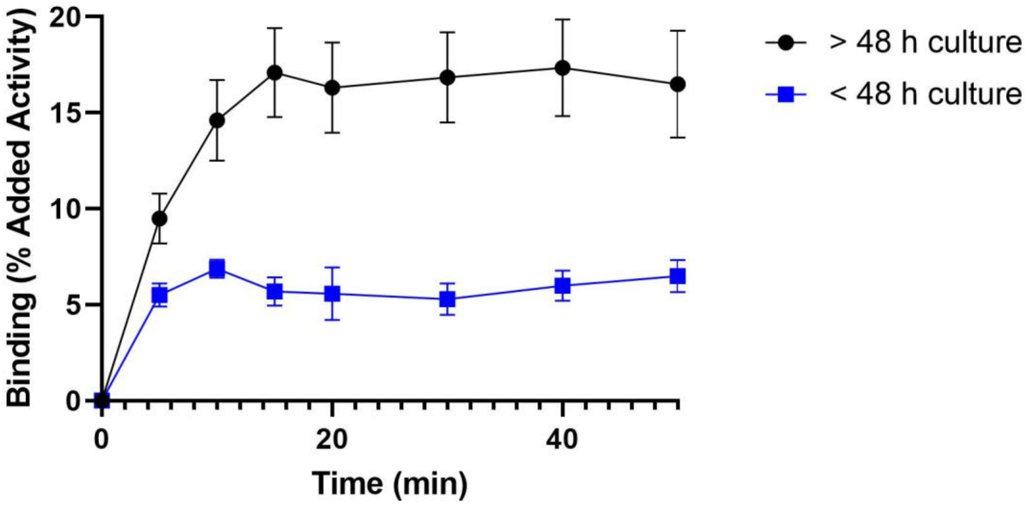


**Figure S6.** Comparison of $[^{11}C]$**5** binding to LNCaP cells cultured for > 48 h (black) and < 48 h (blue). The curves are corrected for decay and non-specific binding, but not corrected for protein content.

## 6. $^1$H and $^{13}$C NMR Spectra of Compounds

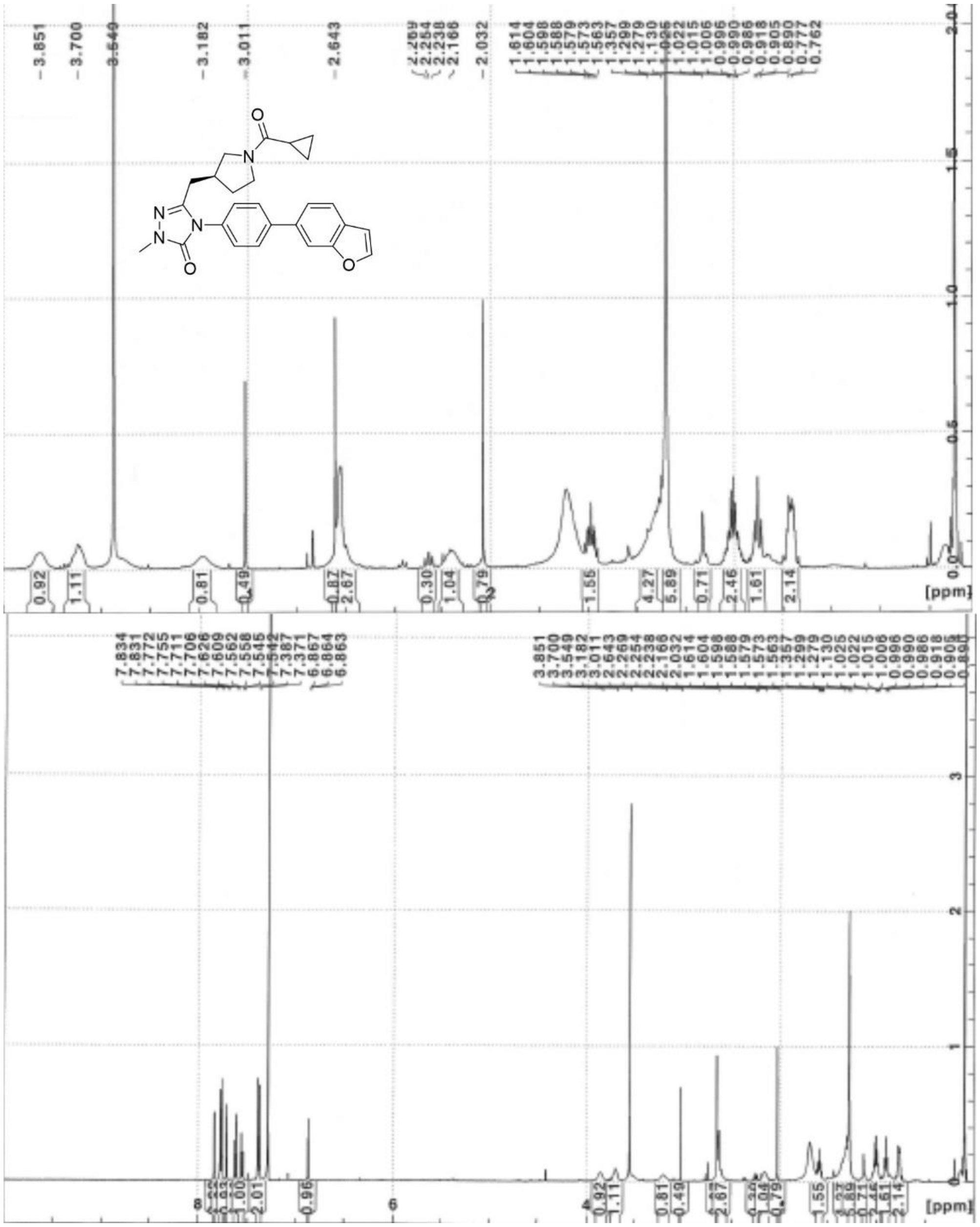

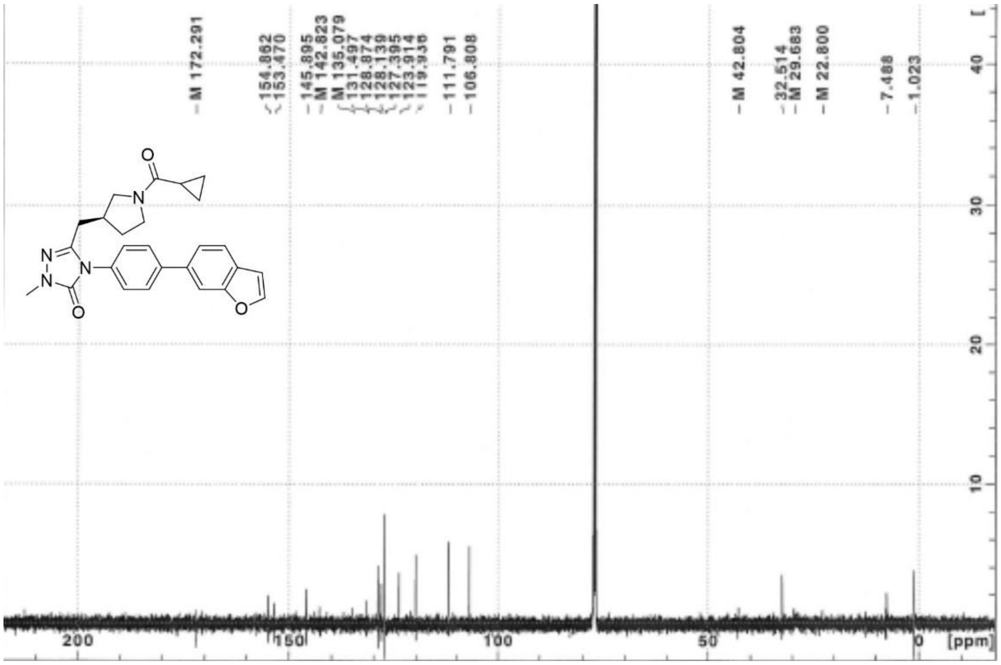
M 172.291
154.862
153.470
145.895
M 142.823
M 135.079
131.497
128.874
128.139
127.395
123.914
119.936
111.791
106.808
M 42.804
32.514
M 29.683
M 22.800
7.488
1.023
200
150
100
50
0
[ppm]

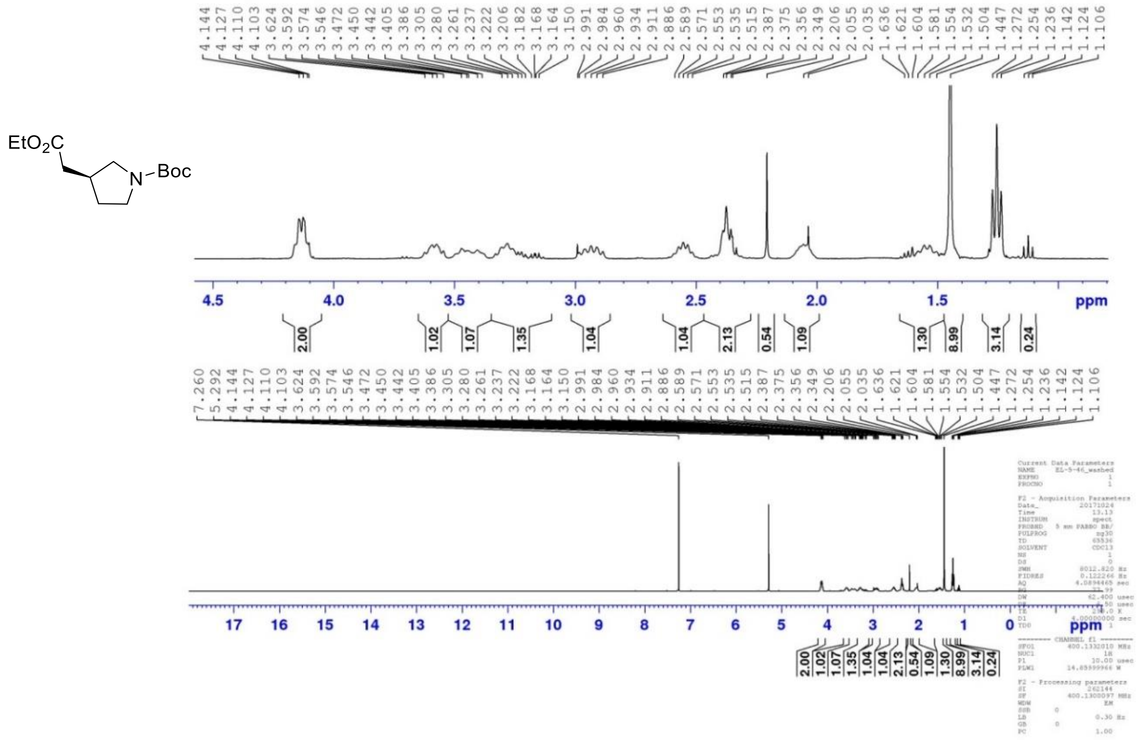
EtO2C
N–Boc
ppm

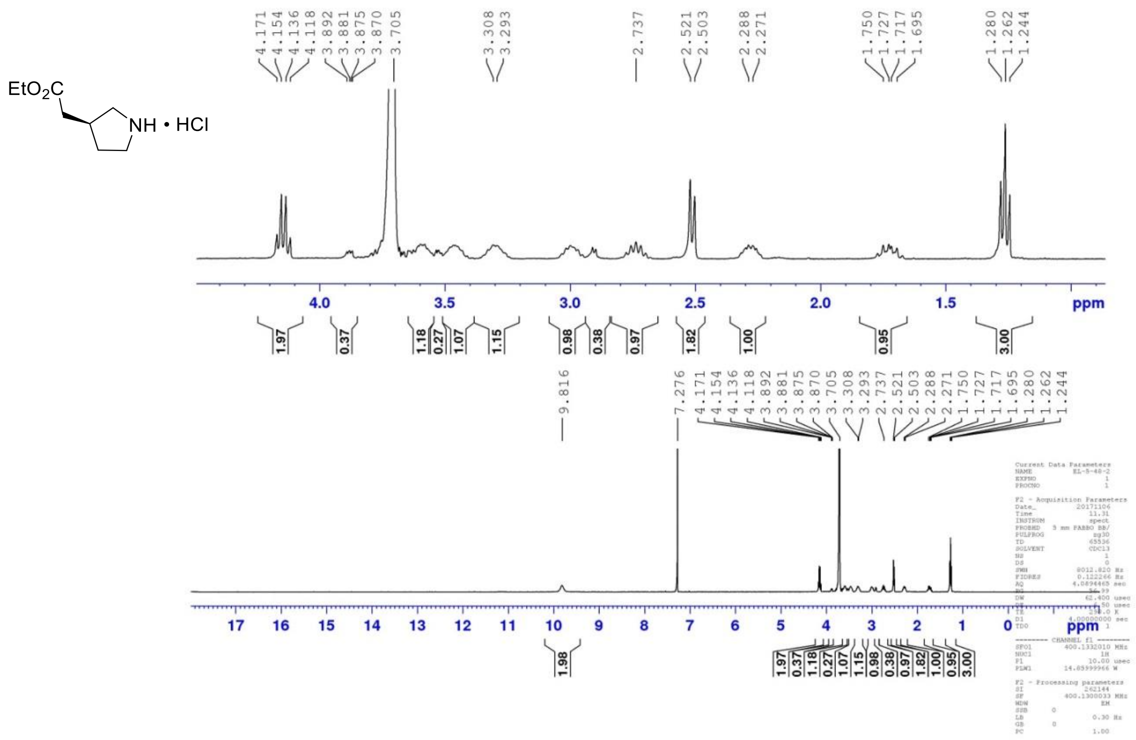
EtO2C
NH • HCl
ppm

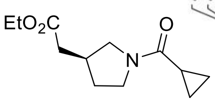

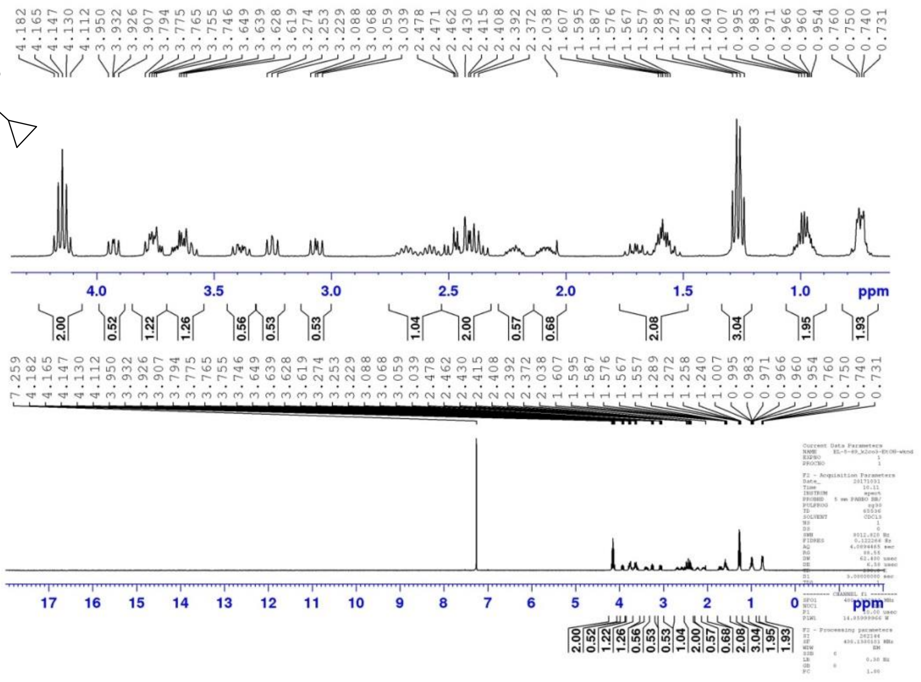

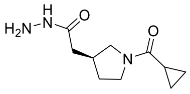
H
$H_2N$
N
O
N
O

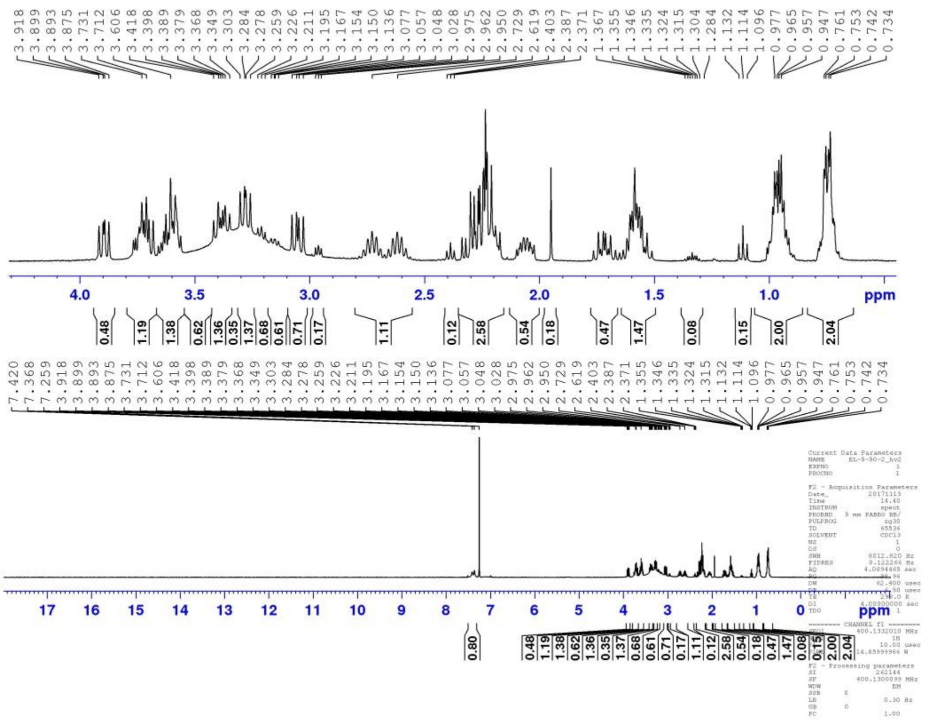
3.918
3.899
3.893
3.875
3.731
3.712
3.606
3.418
3.398
3.389
3.379
3.368
3.349
3.303
3.284
3.278
3.259
3.226
3.211
3.195
3.167
3.154
3.150
3.136
3.077
3.057
3.048
3.028
2.975
2.962
2.950
2.729
2.619
2.403
2.387
2.371
1.367
1.355
1.346
1.335
1.324
1.315
1.304
1.284
1.132
1.114
1.096
0.977
0.965
0.957
0.947
0.761
0.753
0.742
0.734
4.0
3.5
3.0
2.5
2.0
1.5
1.0
ppm
0.48
1.19
1.38
0.62
1.36
0.35
1.37
0.68
0.61
0.71
0.17
1.11
0.12
2.58
0.54
0.18
0.47
1.47
0.08
0.15
2.00
2.04
17 16 15 14 13 12 11 10 9 8 7 6 5 4 3 2 1 0 ppm
0.80

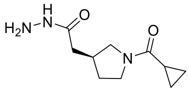
H
$H_2N$
N
O
N
O

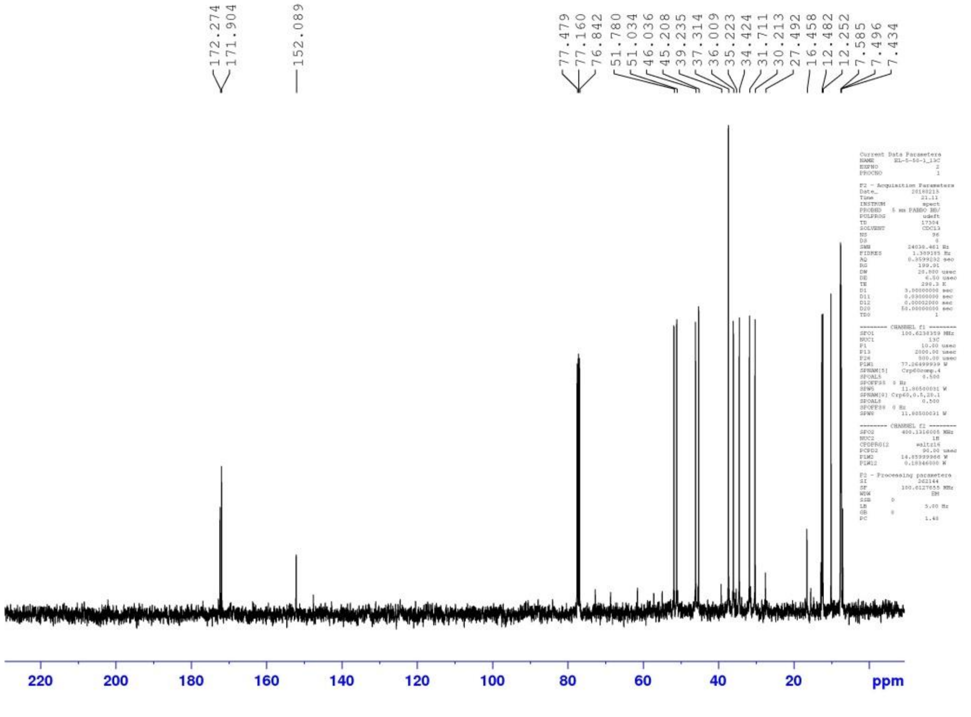
172.274
171.904
152.089
77.479
77.160
76.842
51.780
51.034
46.036
45.208
39.235
37.314
36.009
35.223
34.424
31.711
30.213
27.492
16.458
12.482
12.252
7.585
7.496
7.434
220 200 180 160 140 120 100 80 60 40 20 ppm

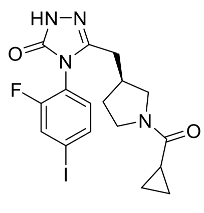
HN–N
O
N
F
I
N
O

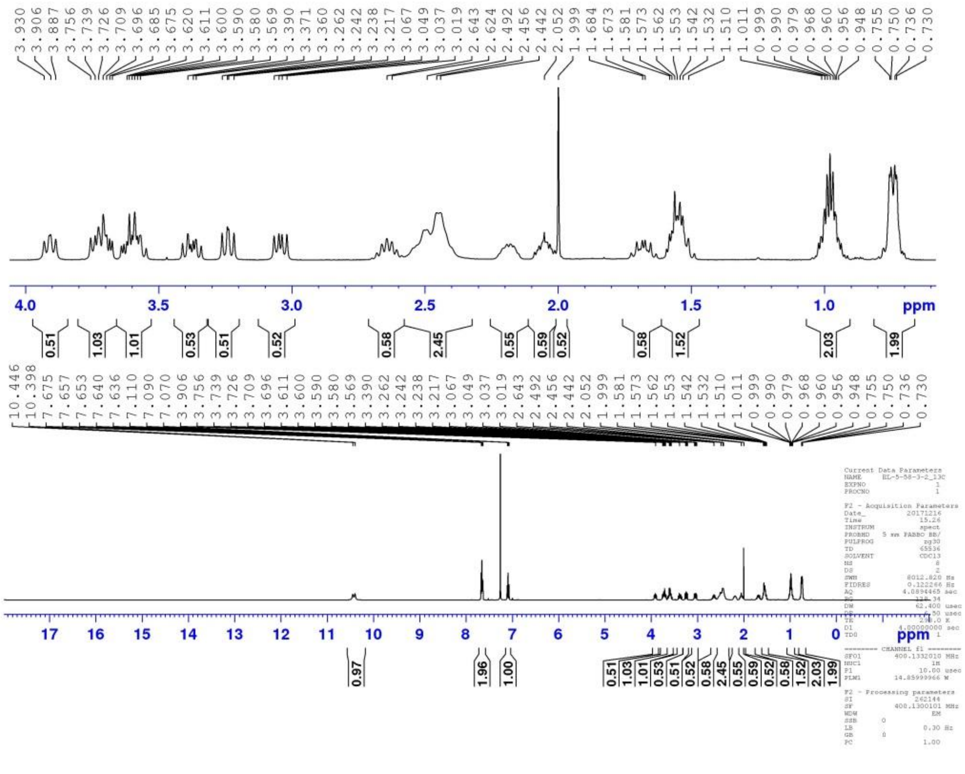
3.930 3.906 3.887 3.756 3.739 3.726 3.709 3.696 3.685 3.675 3.620 3.611 3.600 3.590 3.580 3.569 3.390 3.371 3.360 3.262 3.242 3.238 3.217 3.067 3.049 3.037 3.019 2.643 2.624 2.492 2.456 2.442 2.052 1.999 1.684 1.673 1.581 1.573 1.562 1.553 1.542 1.532 1.510 1.011 0.999 0.990 0.979 0.968 0.960 0.956 0.948 0.755 0.750 0.736 0.730
4.0 3.5 3.0 2.5 2.0 1.5 1.0 ppm
0.51 1.03 1.01 0.53 0.51 0.52 0.58 2.45 0.55 0.59 0.52 0.58 1.52 2.03 1.99
10.446 10.398 7.675 7.657 7.653 7.640 7.636 7.110 7.090 7.070 3.906 3.756 3.739 3.726 3.709 3.696 3.611 3.600 3.590 3.580 3.569 3.390 3.262 3.242 3.238 3.217 3.067 3.049 3.037 3.019 2.643 2.492 2.456 2.442 2.052 1.999 1.581 1.573 1.562 1.553 1.542 1.532 1.510 1.011 0.999 0.990 0.979 0.968 0.960 0.956 0.948 0.755 0.750 0.736 0.730
17 16 15 14 13 12 11 10 9 8 7 6 5 4 3 2 1 0 ppm
0.97 1.96 1.00 0.51 1.03 1.01 0.53 0.51 0.52 0.58 2.45 0.55 0.59 0.52 0.58 1.52 2.03 1.99

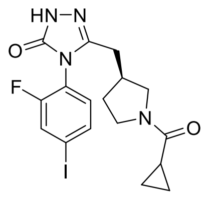
HN–N
O
N
F
I
N
O

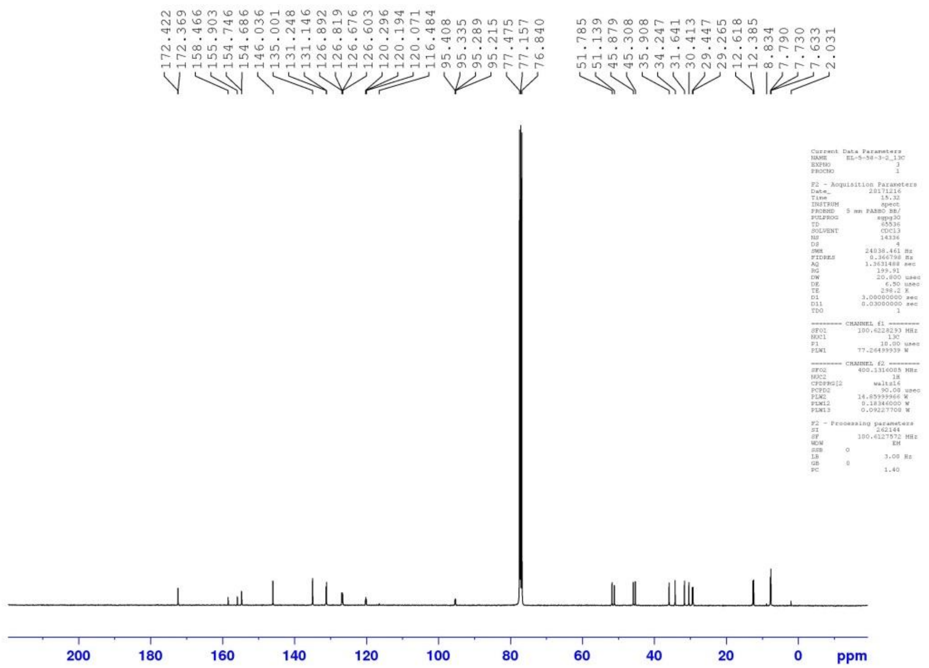
172.422 172.369 158.466 155.903 154.746 154.686 146.036 135.001 131.248 131.146 126.892 126.819 126.676 126.603 120.296 120.194 120.071 116.484 95.408 95.335 95.289 95.215 77.475 77.157 76.840 51.785 51.139 45.879 45.308 35.908 34.247 31.641 30.413 29.447 29.265 12.618 12.385 8.834 7.790 7.730 7.633 2.031
200 180 160 140 120 100 80 60 40 20 0 ppm

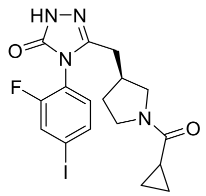
HN–N
O
N
F
N
O
I

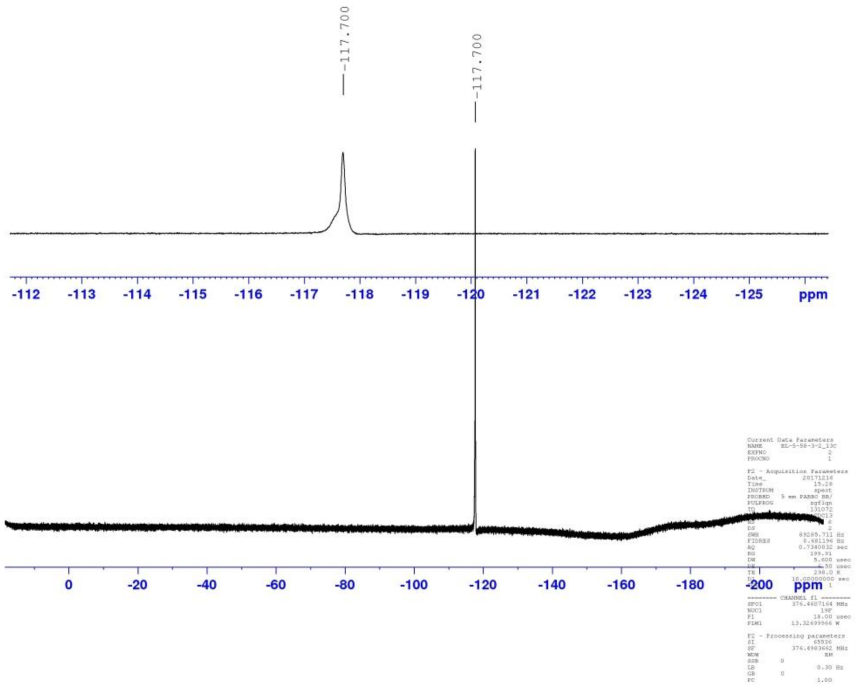
-117.700
-117.700
-112 -113 -114 -115 -116 -117 -118 -119 -120 -121 -122 -123 -124 -125 ppm
0 -20 -40 -60 -80 -100 -120 -140 -160 -180 -200 ppm
Current Data Parameters
NAME EL-5-58-3-2_13C
EXPNO 2
PROCNO 1
F2 - Acquisition Parameters
Date_ 20171216
Time 15.28
INSTRUM spect
PULPROG zgflqn
TD 131072
DS 2
SWH 89285.711 Hz
FIDRES 0.681196 Hz
AQ 0.7340032 sec
RG 199.01
DW 5.600 usec
TE 298.0 K
======== CHANNEL f1 ========
SFO1 376.4607164 MHz
NUC1 19F
P1 18.00 usec
PLW1 13.32699966 W
F2 - Processing parameters
SI 65536
SF 376.4983662 MHz
WDW EM
SSB 0
LB 0.30 Hz
GB 0
PC 1.00

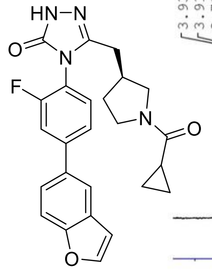
HN–N
O
N
F
N
O
O

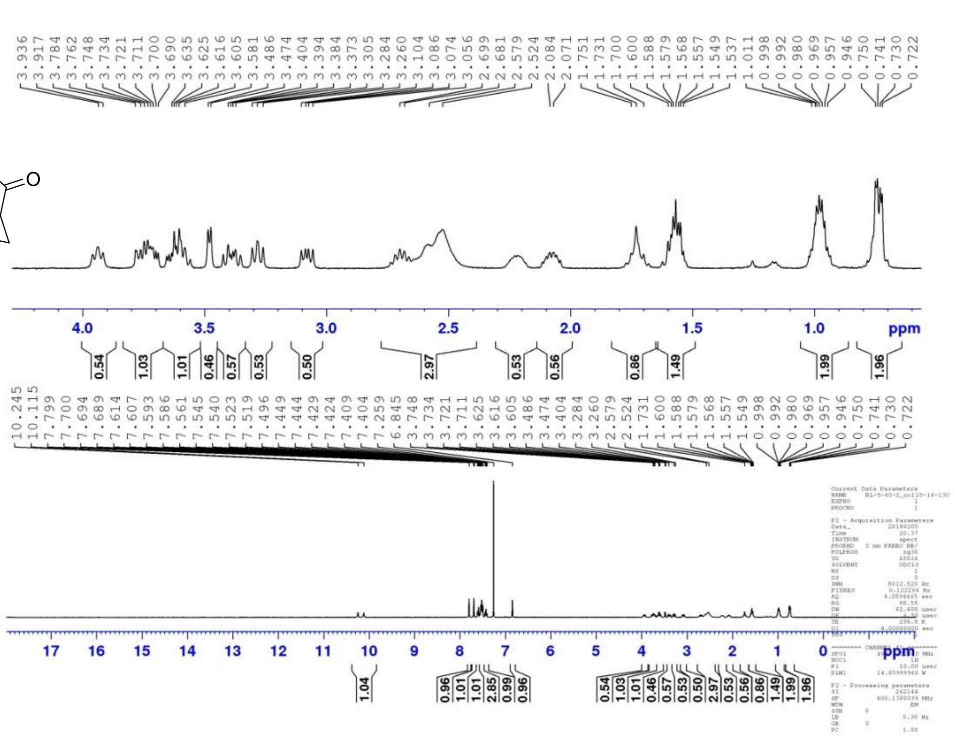
3.936 3.917 3.784 3.762 3.748 3.734 3.721 3.711 3.700 3.690 3.635 3.625 3.616 3.605 3.581 3.486 3.474 3.404 3.394 3.384 3.373 3.305 3.284 3.260 3.104 3.086 3.074 3.056 2.699 2.681 2.579 2.524 2.084 2.071 1.751 1.731 1.700 1.600 1.588 1.579 1.568 1.557 1.549 1.537 1.011 0.998 0.992 0.980 0.969 0.957 0.946 0.750 0.741 0.730 0.722
4.0 3.5 3.0 2.5 2.0 1.5 1.0 ppm
0.54 1.03 1.01 0.46 0.57 0.53 0.50 2.97 0.53 0.56 0.86 1.49 1.99 1.96
10.245 10.115 7.799 7.700 7.694 7.689 7.614 7.607 7.593 7.586 7.561 7.545 7.540 7.523 7.519 7.496 7.449 7.444 7.429 7.424 7.409 7.404 7.259 6.845 3.748 3.734 3.721 3.711 3.625 3.616 3.605 3.486 3.474 3.404 3.284 3.260 2.579 2.524 1.731 1.600 1.588 1.579 1.568 1.557 1.549 0.998 0.992 0.980 0.957 0.946 0.750 0.741 0.730 0.722
17 16 15 14 13 12 11 10 9 8 7 6 5 4 3 2 1 0 ppm
1.04 0.96 1.01 1.01 2.85 0.99 0.96 0.54 1.03 1.01 0.46 0.57 0.53 0.50 2.97 0.53 0.56 0.86 1.49 1.99 1.96

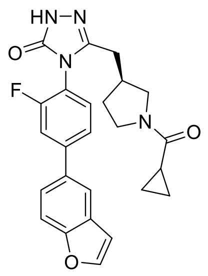
HN–N
O
N
F
N
O
O

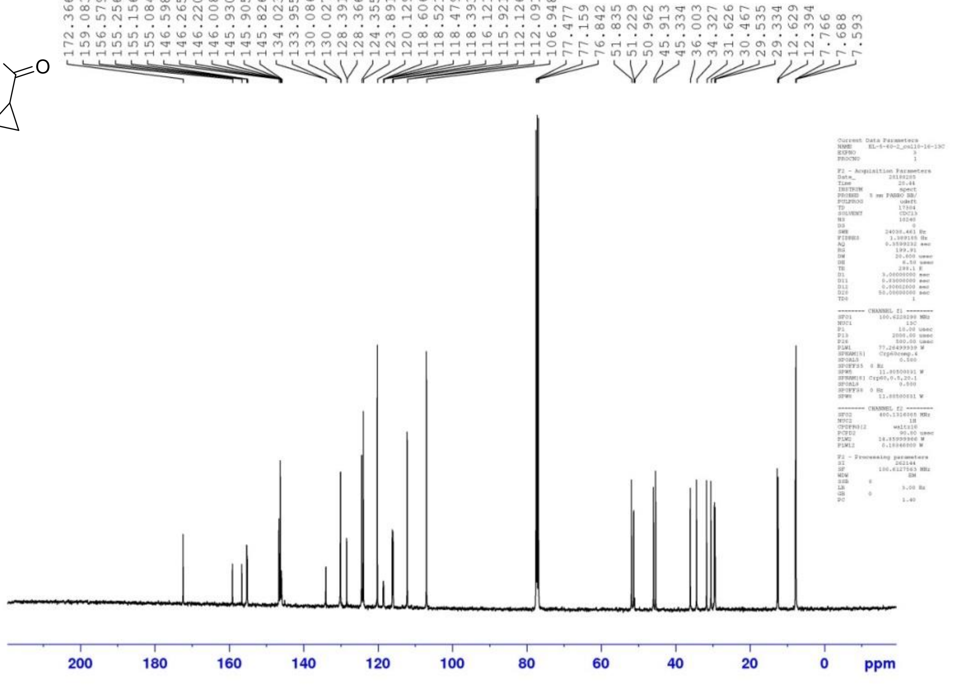
172.366 159.083 156.579 155.256 155.156 155.084 146.598 146.265 146.220 146.008 145.930 145.905 145.826 134.023 133.955 130.086 130.027 128.391 128.366 124.355 123.891 120.129 118.606 118.521 118.479 118.393 116.121 115.921 112.126 112.091 106.948 77.477 77.159 76.842 51.835 51.229 50.962 45.913 45.334 36.003 34.327 31.626 30.467 29.535 29.334 12.629 12.394 7.766 7.688 7.593
200 180 160 140 120 100 80 60 40 20 0 ppm

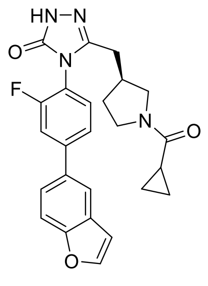
HN–N
O
N
F
N
O
O

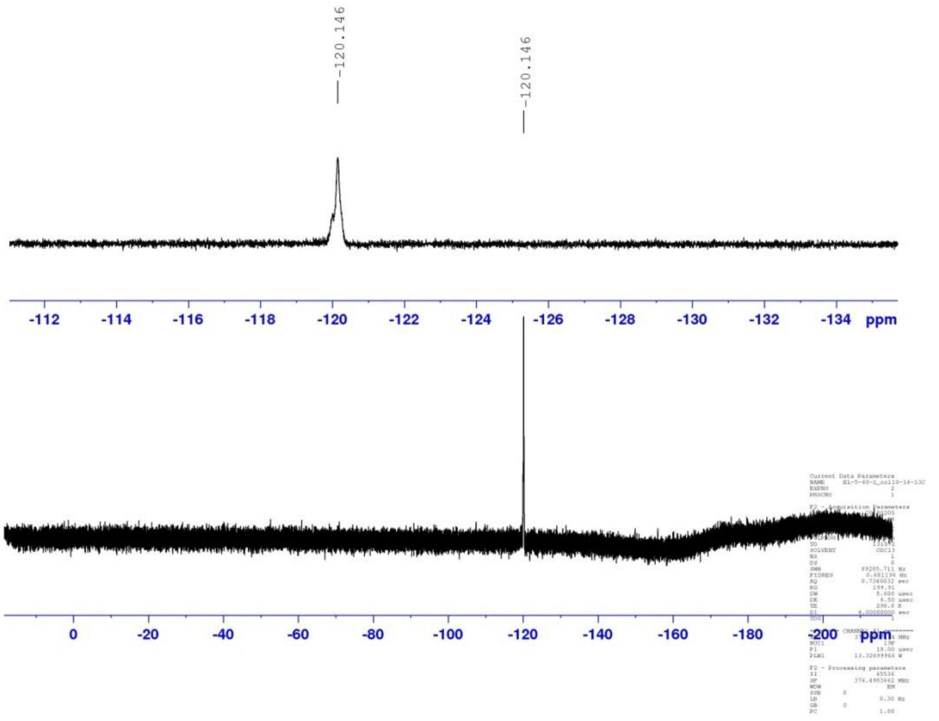
-120.146
-120.146
-112 -114 -116 -118 -120 -122 -124 -126 -128 -130 -132 -134 ppm
0 -20 -40 -60 -80 -100 -120 -140 -160 -180 -200 ppm

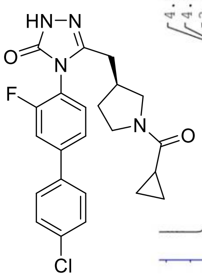

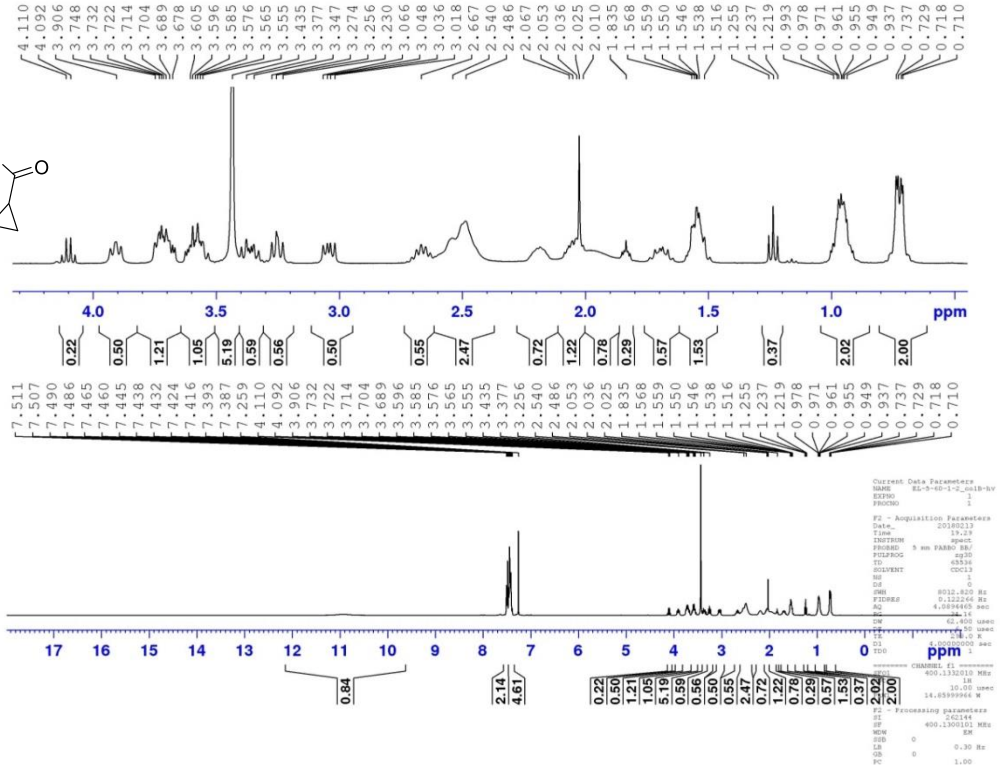

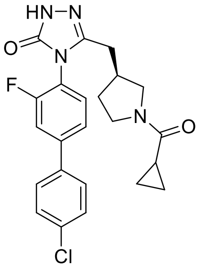

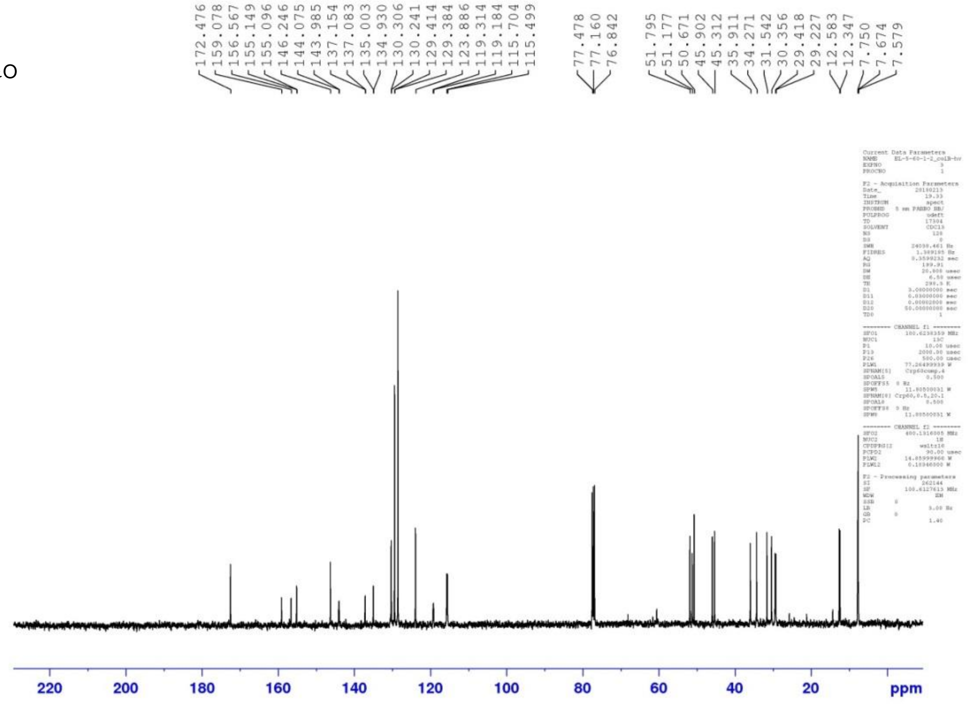

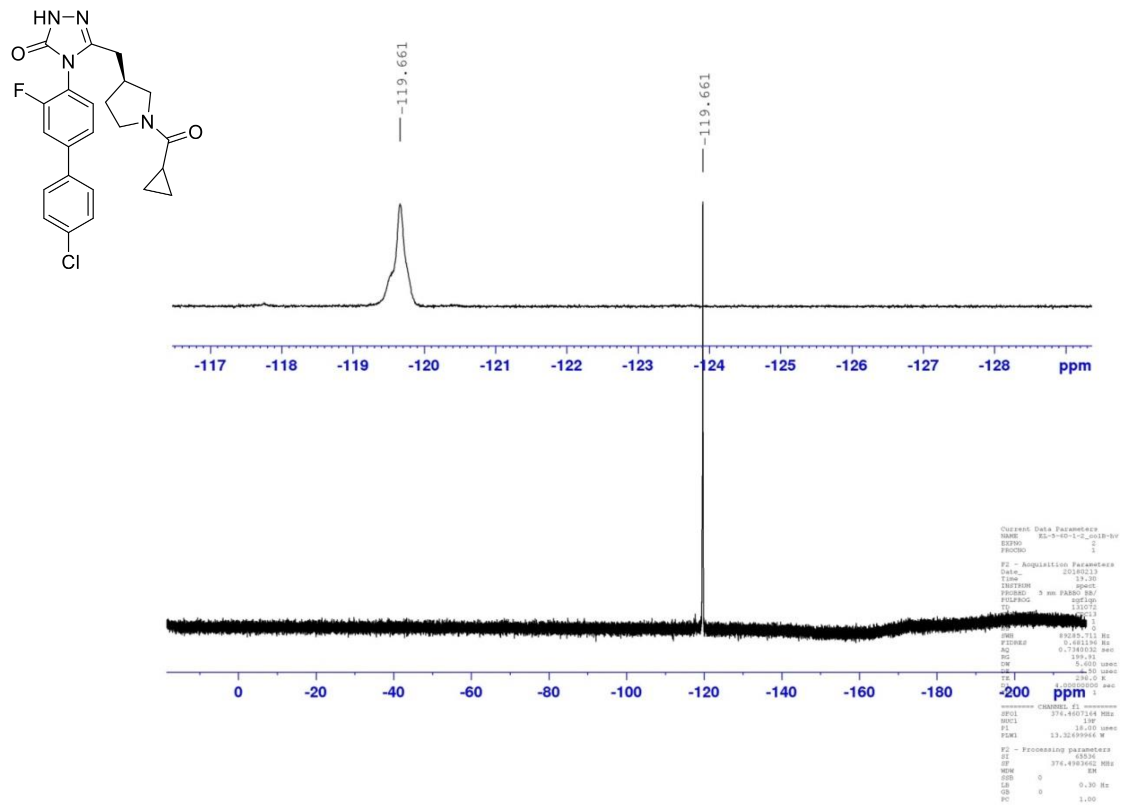
HN–N
O
N
F
N
O
Cl
-119.661
-119.661
-117 -118 -119 -120 -121 -122 -123 -124 -125 -126 -127 -128 ppm
0 -20 -40 -60 -80 -100 -120 -140 -160 -180 -200 ppm

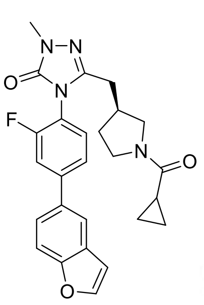

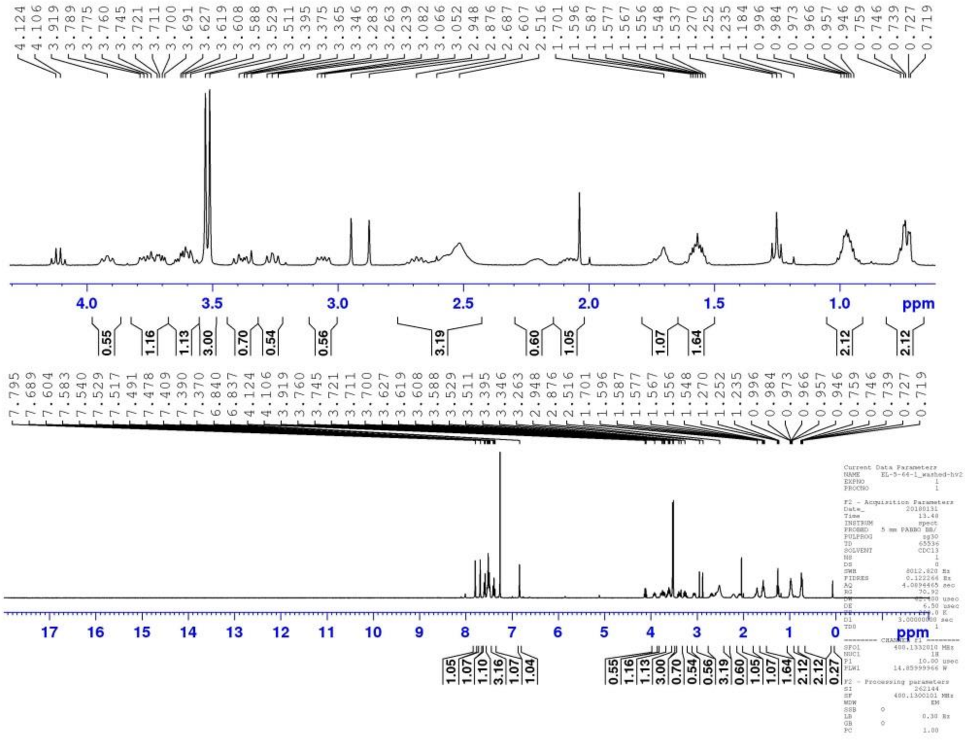

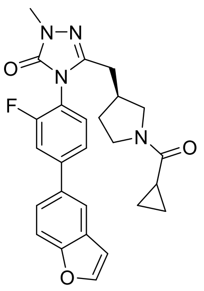

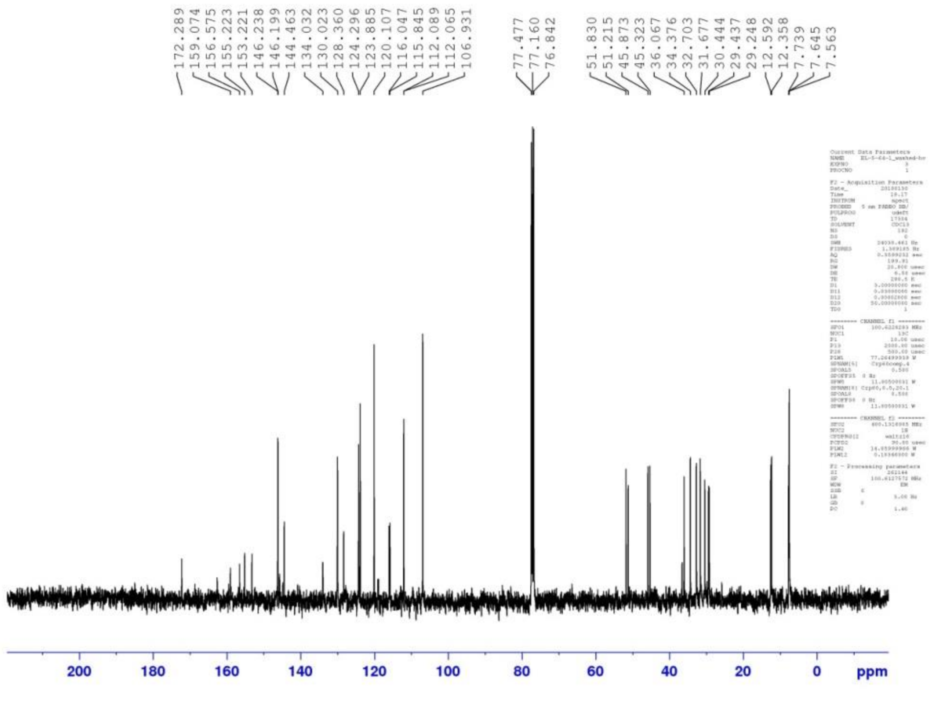

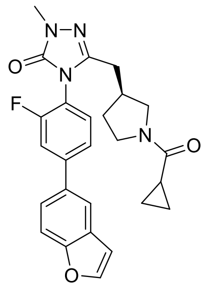
N
N
O
N
F
N
O
O

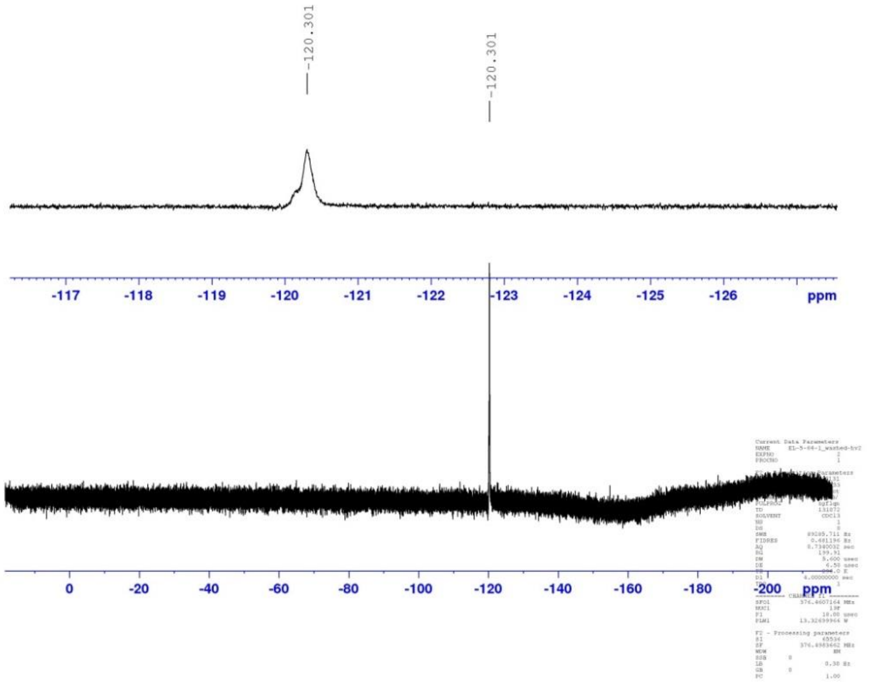
-120.301
-120.301
-117
-118
-119
-120
-121
-122
-123
-124
-125
-126
ppm
0
-20
-40
-60
-80
-100
-120
-140
-160
-180
-200
ppm

**7.**

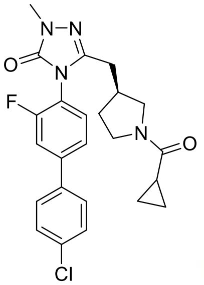
N
N
O
N
F
N
O
Cl

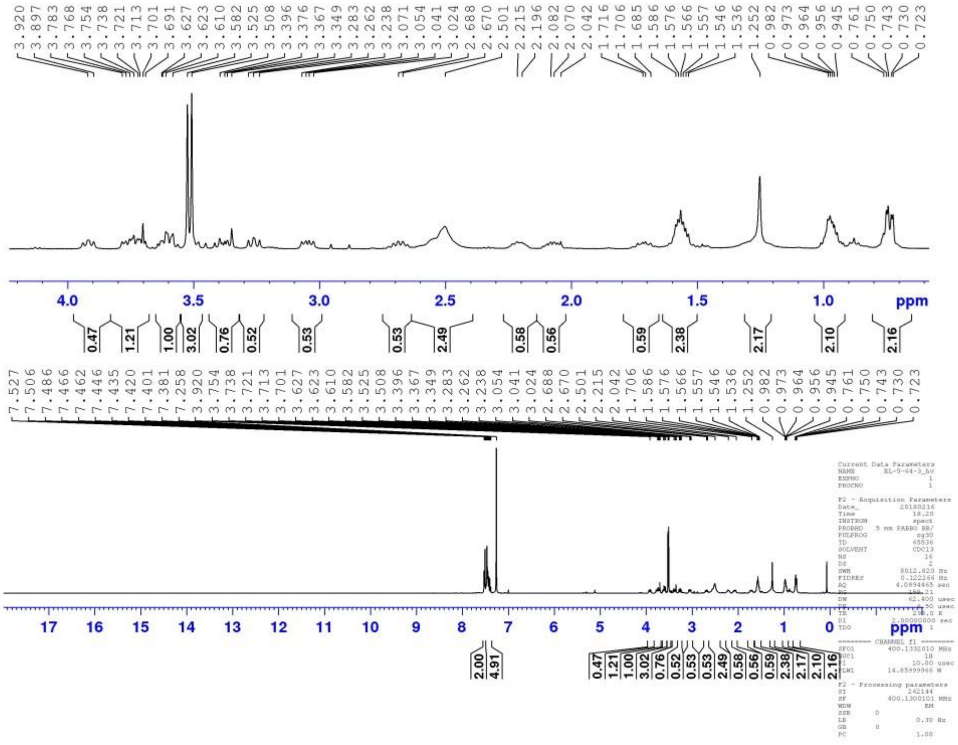
3.920 3.897 3.783 3.768 3.754 3.738 3.721 3.713 3.701 3.691 3.627 3.623 3.610 3.582 3.525 3.508 3.396 3.376 3.367 3.349 3.283 3.262 3.238 3.071 3.054 3.041 3.024 2.688 2.670 2.501 2.215 2.196 2.082 2.070 2.042 1.716 1.706 1.685 1.586 1.576 1.566 1.557 1.546 1.536 1.252 0.982 0.973 0.964 0.956 0.945 0.761 0.750 0.743 0.730 0.723
4.0
3.5
3.0
2.5
2.0
1.5
1.0
ppm
0.47 1.21 1.00 3.02 0.76 0.52 0.53 0.53 2.49 0.58 0.56 0.59 2.38 2.17 2.10 2.16
7.527 7.506 7.486 7.462 7.446 7.435 7.420 7.401 7.381 7.258 3.920 3.754 3.738 3.721 3.713 3.701 3.627 3.623 3.610 3.582 3.525 3.508 3.396 3.367 3.349 3.283 3.262 3.238 3.054 3.041 3.024 2.670 2.501 2.215 2.042 1.706 1.586 1.576 1.566 1.557 1.546 1.536 1.252 0.982 0.973 0.964 0.956 0.945 0.761 0.750 0.743 0.730 0.723
17 16 15 14 13 12 11 10 9 8 7 6 5 4 3 2 1 0 ppm
2.00 4.91
0.47 1.21 1.00 3.02 0.76 0.52 0.53 0.53 2.49 0.56 0.58 0.59 2.38 2.17 2.10 2.16

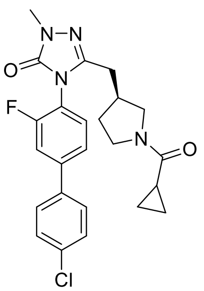
N
N
O
N
F
N
O
Cl

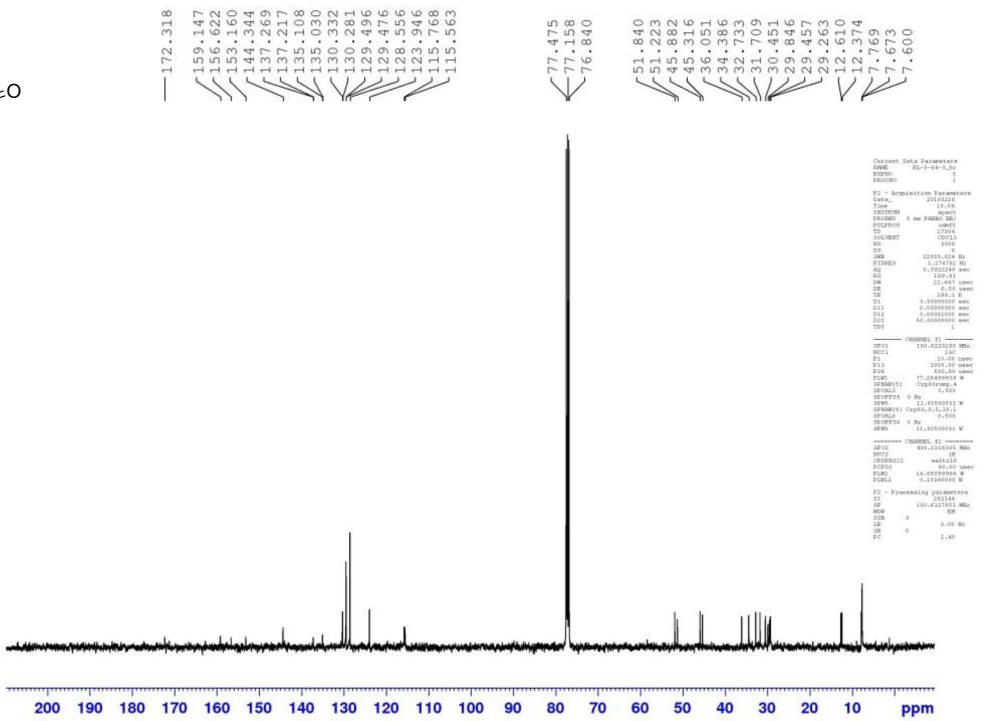
172.318
159.147 156.622 153.160 144.344 137.269 137.217 135.108 135.030 130.332 130.281 129.496 129.476 128.556 123.946 115.768 115.563
77.475 77.158 76.840
51.840 51.223 45.882 45.316 36.051 34.386 32.733 31.709 30.451 29.846 29.457 29.263 12.610 12.374 7.769 7.673 7.600
200 190 180 170 160 150 140 130 120 110 100 90 80 70 60 50 40 30 20 10 ppm

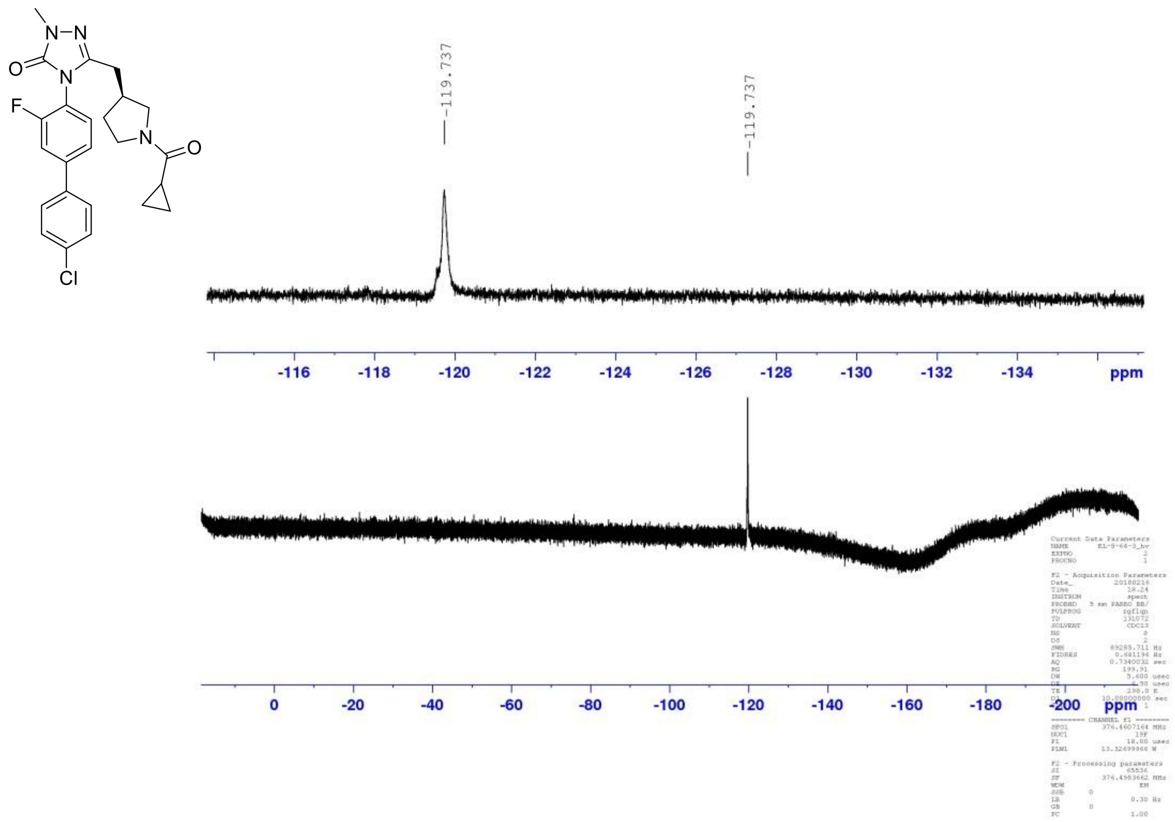
N
N
O
N
F
N
O
Cl
-119.737
-119.737
-116
-118
-120
-122
-124
-126
-128
-130
-132
-134
ppm
0
-20
-40
-60
-80
-100
-120
-140
-160
-180
-200
ppm
